# Identification of Salicylates in Willow Bark (*Salix* Cortex) for Targeting Peripheral Inflammation

**DOI:** 10.3390/ijms222011138

**Published:** 2021-10-15

**Authors:** Kyriaki Antoniadou, Corinna Herz, Nguyen Phan Khoi Le, Verena Karolin Mittermeier-Kleßinger, Nadja Förster, Matthias Zander, Christian Ulrichs, Inga Mewis, Thomas Hofmann, Corinna Dawid, Evelyn Lamy

**Affiliations:** 1 Food Chemistry and Molecular Sensory Science, Technical University of Munich, 85354 Freising, Germany; kyriaki.antoniadou@tum.de (K.A.); verena.mittermeier@tum.de (V.K.M.-K.); thomas.hofmann@tum.de (T.H.); 2 Molecular Preventive Medicine, University Medical Center and Faculty of Medicine, University of Freiburg, 79108 Freiburg, Germany; corinna.herz@uniklinik-freiburg.de (C.H.); phan.khoi.nguyen.le@uniklinik-freiburg.de (N.P.K.L.); 3 Urban Plant Ecophysiology, Humboldt University of Berlin, 14195 Berlin, Germany; nadja.foerster@hu-berlin.de (N.F.); matthias.zander@hu-berlin.de (M.Z.); christian.ulrichs@hu-berlin.de (C.U.); inga@entomology.de (I.M.)

**Keywords:** *Salix*, willow bark extract, phytopharmaceutical, salicylates, catechol, anti-inflammation, herbal medicine, pain

## Abstract

*Salix* cortex-containing medicine is used against pain conditions, fever, headaches, and inflammation, which are partly mediated via arachidonic acid-derived prostaglandins (PGs). We used an activity-guided fractionation strategy, followed by structure elucidation experiments using LC-MS/MS, CD-spectroscopy, and 1D/2D NMR techniques, to identify the compounds relevant for the inhibition of PGE_2_ release from activated human peripheral blood mononuclear cells. Subsequent compound purification by means of preparative and semipreparative HPLC revealed 2′-*O*-acetylsalicortin (**1**), 3′-*O*-acetylsalicortin (**2**), 2′-*O*-acetylsalicin (**3**), 2′,6′-*O*-diacetylsalicortin (**4**), lasiandrin (**5**), tremulacin (**6**), and cinnamrutinose A (**7**). In contrast to **3** and **7**, compounds **1**, **2**, **4**, **5**, and **6** showed inhibitory activity against PGE_2_ release with different potencies. Polyphenols were not relevant for the bioactivity of the *Salix* extract but salicylates, which degrade to, e.g., catechol, salicylic acid, salicin, and/or 1-hydroxy-6-oxo-2-cycohexenecarboxylate. Inflammation presents an important therapeutic target for pharmacological interventions; thus, the identification of relevant key drugs in *Salix* could provide new prospects for the improvement and standardization of existing clinical medicine.

## 1. Introduction

Using medicinal plants for the modulation of inflammation, especially in the suppression of inflammation, can be a relevant alternate to conventional therapeutic strategies [1]. The bark of the willow tree (*Salix* spp.), which belongs to the Salicaceae family, is used in phytopharmaceutical products against pain conditions, fever, headaches, or inflammation [2]. Extracts of the *Salix* cortex have a high content of phenolic glycosides [3], with up to 30% plant dry weight [4]. Salicylates are described as the main group within these phenolic glycosides [5], and salicin has been described in the past as the active constituent of the bark extract [6,7]. Thus, pharmaceutical drugs from willow bark are standardized to salicylic derivatives, expressed as salicin [2]. However, pharmacological studies have shown that the clinical efficacy of a willow bark extract cannot be explained by its salicin content alone. As an example, on a mg/kg basis, a standardized willow bark extract (STW 33-I, Steigerwald Arzneimittelwerk Gmbh, Darmstadt, Germany) was comparably effective in reducing inflammation as acetyl salicylic acid (ASA) [8]. The extract contained only 24% salicin; thus, its total activity was likely dependent on even more substances. Polyphenols have been discussed as an important factor here [9]. Besides salicylic acid identified from salicin [10], in 2013, Knuth and colleagues identified catechol as another important in vivo metabolite of willow bark compounds containing a 1-hydroxy-6-oxo-2-cycohexenecarboxylate (HCH) moiety, such as salicortin [11]. Catechol is known to inhibit different inflammatory markers in vitro [12,13,14] and has consequently been proposed to contribute to the analgesic and anti-inflammatory potency of willow bark [11]. Besides the need for the further identification of active compounds, another challenge in the use of willow bark as a pharmaceutical remedy is that, depending on the genotype, the distribution of phenolic glycosides and the total polyphenolic content may show large variations [3,15,16,17]. This is very likely another reason for the inconsistent and unpredictable therapeutic efficacy of the bark extract, which currently hinders product optimization. In screening studies of our group on different *Salix* species, we recently identified extracts from *S. pentandra* as the most effective ones against inflammatory pain *in vitro* (data unpublished). According to the study by Förster, Ulrichs [3], *S. daphnoides* and *S. purpurea* contained predominantly salicylate salicortin, whereas 2-*O*-acetylsalicortin (**1**) was present at higher amounts in *S. pentandra*. Ruuhola and Julkunen-Tiitto [18] and Ruuhola, Julkunen-Tiitto, and P. Vainiotalo [19] postulated that *S. pentandra* contained not only (non)acetylated salicortin (**1** and **2**) but, also, salicin, acetyltremulacin, and tremulacin (**6**). Furthermore, a previous work on *S. pentandra* bark material enabled the spectroscopic identification of **1**, **3**, grandidentatin, and ampelopsin [20].

The arachidonic acid-cyclooxygenase (COX)-prostaglandin (PGs) signaling pathway has an important modulatory role on inflammatory pain conditions, fever, and headaches [21,22]. Pharmacological interventions upstream of PGE_2_ (prostaglandin E_2_) may greatly interfere with proinflammatory and pronociceptive actions [23,24]. One of the analgesic effects of aspirin in arthritis is attributed to the peripheral inhibition of PGs at the inflamed site [25]. The aim of the study was, thus, to systematically identify compounds from *S. pentandra* with inhibitory potential against PGE_2_ release from lipopolysaccharide (LPS)-stimulated primary human peripheral blood mononuclear cells (PBMC) using bioactivity-guided fractionation. Furthermore, whole extracts and their corresponding solid-phase extraction (SPE) fractions on COX-1 and COX-2 enzyme inhibition were assessed.

## 2. Results and Discussion

### 2.1. Activity-Guided Fractionation of Salix Bark Extract

**Figure 1:**
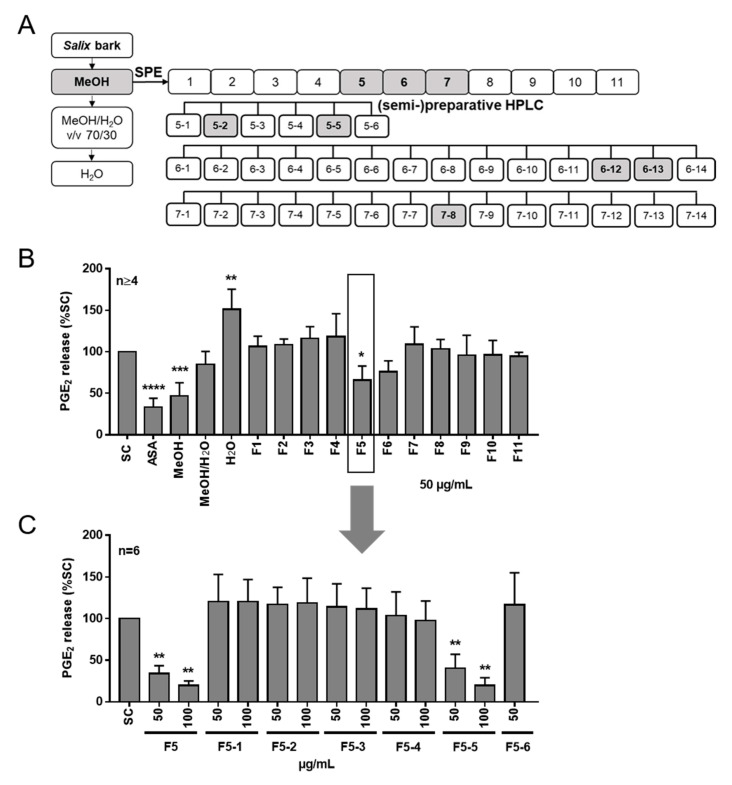
Fractionation scheme and bioactivity of *S. pentandra* bark extracts and solid-phase extraction fractions. (**A**) The bioactive methanol extract was fractionated using SPE. SPE fraction F5 was further separated by means of (semi)preparative HPLC, and the compounds were subsequently purified and isolated. Grey labeling highlights the extract, SPE fractions, and HPLC fractions from which the purified compounds were isolated. (**B**,**C**) Inhibition of PGE_2_ release from activated human PBMC. Cells were stimulated with LPS for 24 h after a 30 min pretreatment with the extracts, SPE fractions, and subfractions of SPE fraction F5 (F5-1 to F5-6, (**C**)) from *S. pentandra*. Data are the means ± standard deviation (SD.) Asterisks indicate statistically significant differences between the respective treatment and the solvent control. * *p* < 0.05, ** *p* < 0.01, *** *p* < 0.001, and **** *p* < 0.0001. SC = solvent control (1% dd water, (**B**) and 0.1% DMSO, (**C**)). As a reference compound, 1-µg/mL acetyl salicylic acid (ASA) was used (**B**).

In order to investigate the anti-inflammatory potential of *S. pentandra*, sequential solvent fractionation using methanol (yield of 29.57 g/100 g), methanol/water (*v*/*v*, 70/30; yield of 2.3 g/100 g), and water (yield of 2.23 g/100 g) was performed (Figure 1A) to obtain water and/or the solvent soluble compounds. As shown in Figure 1B, the methanol extract inhibited significantly the PGE_2_ release in LPS (lipopolysaccharide)-stimulated human PBMC (peripheral blood mononuclear cells) from healthy donors by 55% compared to the solvent control. In contrast, the methanol/water extract did not inhibit the PGE_2_ release, and the water extract even enhanced the PGE_2_ level.

Subsequently, the bioactive methanol extract was fractionated by means of solid-phase extraction (SPE) into eleven SPE fractions (Figure 1A), and the prostaglandin (PGE_2_) release in LPS-stimulated PBMC was again quantified. As a reference, 1 µg/mL ASA was used. Among the eleven fractions, SPE fractions F5 and F6 showed the highest anti-inflammatory activity, but only fraction F5 achieved statistical significance (*p* < 0.05) (Figure 1B).

**Figure 2:**
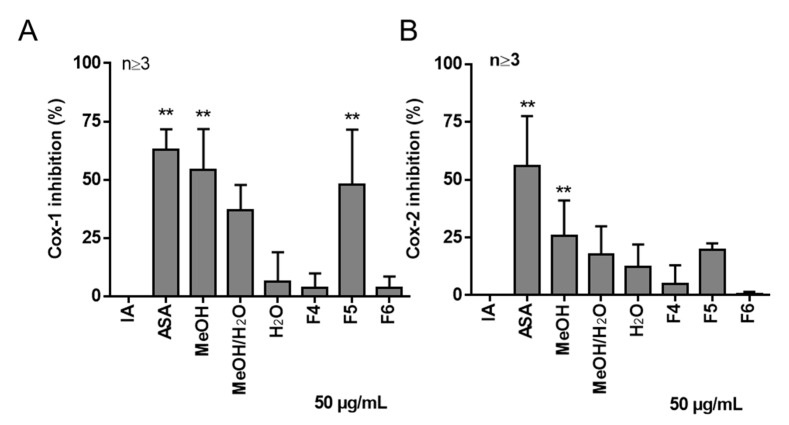
Effects of *Salix* extracts and SPE fractions on COX-1/2 enzyme activity. Human recombinant COX-1 (**A**) or COX-2 (**B**) enzyme activity was analyzed based on the quantification of prostaglandin PGF2α (formed by the SnCl_2_ reduction of COX-derived PGH_2_) using an ELISA (Enzyme-Linked Immunosorbent Assay) assay. Inhibition (%) was calculated by comparison to the initial activity (IA) of the COX-1 or COX-2 protein. ASA, acetyl salicylic acid. Bars are the means + SD. Asterisks indicate statistically significant differences between the respective treatment and the solvent control. ** *p* < 0.01.

Furthermore, we analyzed the effect of *Salix* cortex extracts and SPE fractions on cyclooxygenase isoform 1 (COX-1) and 2 (COX-2) enzyme activity in comparison to ASA, because COX-1 and COX-2 are the key enzymes in PG biosynthesis [26]. Particularly, the therapeutic mode of action of ASA is recognized via inhibiting COX-1 and COX-2, wherein COX-2 converts endogenous AA to PGE_2_ in human PBMC [27]. In contrast to the water extract, the methanol and methanol/water extracts inhibited COX-1 and COX-2 enzyme activity at 50 µg/mL (Figure 2). However, ASA revealed a higher inhibitory potential against COX-2 in contrast to the *Salix* extracts. Recently, it has been already shown by our group that a methanolic *S. pentandra* cortex extract (S6 clone), but no other tested *Salix* cortex extract, blocked COX-1 and COX-2 enzyme activities [28]. The inhibitory potential of S6 in this study was greater compared to the present study. This may be explained by extract standardization of the total phenolic glycosides [28]. SPE fraction F5 showed an inhibitory effect of 47% and 17% of COX-1 and COX-2 enzyme activity, respectively, comparable to the initial methanol extract of *S. pentandra*. The SPE fractions F4 and F6 were also analyzed to investigate any compound carryover between these fractions in comparison to SPE fraction F5 and with regards to their bioactivity. However, the adjacent SPE fractions F4 and F6 did not inhibit COX-1/2 activity. It was shown previously by us that 2′-*O*-acetylsalicortin (**1**), one compound present in SPE fraction F5, did not inhibit the enzyme activity of COX-1/2 [28]. Thus, other compounds eluting in SPE fraction F5 are mainly responsible for the observed enzyme activity, which should be investigated in further studies.

**Figure 3:**
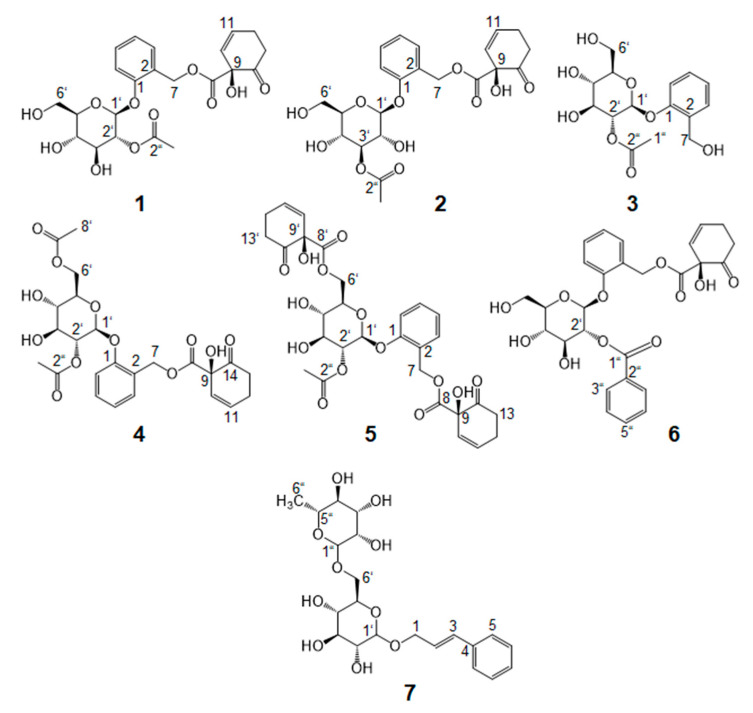
Chemical structures of isolated salicylates from the methanol extract of *S. pentandra* bark: 2′-*O*-acetylsalicortin (**1**), 3′-*O*-acetylsalicortin (**2**), 2′-*O*-acetylsalicin (**3**), 2′,6′-*O*-diacetylsalicortin (**4**), lasiandrin (**5**), tremulacin (**6**), and a non-salicylate cinnamrutinose A (**7**).

Consequently, the most potent SPE fraction F5 was further separated chromatographically into six subfractions (F5-1 to F5-6) by means of preparative HPLC (Supplementary Material Figure S1). Only fraction F5-5 blocked PGE_2_ release in LPS-stimulated PBMC in a similar efficacy as SPE fraction F5 (Figure 1C). To identify the bioactive compounds that mediate the anti-inflammatory potential of fraction F5-5, further purification steps were performed by chromatographic separation techniques. Chemical structures of isolated salicylates from the methanol extract of *S. pentandra* bark are shown in Figure 3. First, single compounds **1**, **2**, and **3** were isolated and purified using semipreparative HPLC (Supplementary Material Figure S2), and the structures were elucidated by means of UPLC-ToF-MS (Supplementary Material Figures S3–S5) and 1D/2D-NMR (Supplementary Material Tables S1–S3).

**Figure 4:**
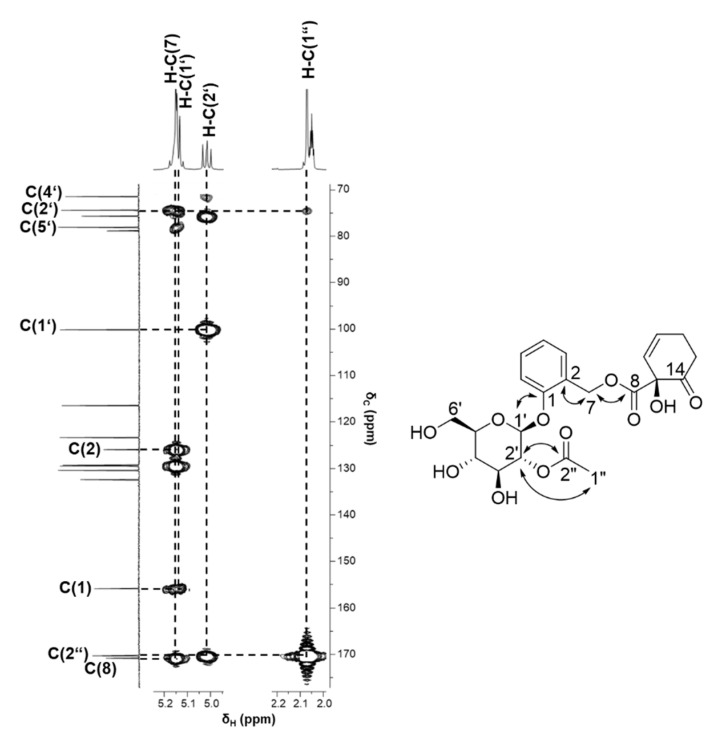
Heteronuclear multiple bond correlation (HMBC) of **1**, indicating the correlations between the sugar unit with the acetyl group and the phenolic ring, as well as the coupling between the methylene protons H-C(7) and the carbonyl carbon atom C(8).

2′-*O*-acetylsalicortin (**1**), purified from fraction F5-5-7, was analyzed by means of UPLC-ToF-MS in the negative ionization mode, revealing a pseudo molecular ion *m*/*z* 465.1434 ([M-H]^-^) and indicating a molecular formula of C_22_H_26_O_11_. The UV maxima of 220 and 272 nm were indicative of the existence of a phenolic ring, which was also verified by ^1^H-NMR signals (*δ*_H_): 7.31 ppm (H-C(3)), 7.05 ppm (H-C(4)), 7.28 ppm (H-C(5)), and 7.21 ppm (H-C(6)). The coupling constants of ^4^*J*_H,H_ = 7.50 and 8.0 Hz for H-C(4) and H-C(6), as well as the multiplicity of H-C(3) and H-C(5), confirmed the ortho substitution. The heteronuclear couplings of carbon C(1) at 155.88 ppm and the *β*-anomeric proton H-C(1′) at 5.13 ppm (d, ^3^*J*_C,H_ = 7.16 Hz) of *D*-glucose confirmed the connection of the sugar moiety to the phenolic ring (Figure 4). Homo- and heteronuclear couplings of proton H-C(2′) at 5.02 ppm to H-C(1″) observed at 2.07 ppm and C(2″) and resonating at 170.30 ppm confirmed the acetylation. Moreover, protons H-C(7) at 5.18 ppm of the methylene group were coupled with the quaternary carbon atom C(2) (125.94 ppm) and the carbonyl carbon atom C(8) (170.79 ppm) (Figure 4), indicating the coupling of the phenol group with the 1-hydroxy-6-oxo-2-cycohexenecarboxylate (HCH) group. By means of the COSY experiment, proton–proton correlations could be assigned between the double bond H-C(10)/H-C(11) and two methylene groups, H-C(12) and H-C(13), as part of the HCH moiety. The identified coupling constant ^3^*J*_H,H_ = 9.70 Hz for both H-C(10) and H-C(11) was indicative of the *cis*-configuration of the salicylate. Moreover, the carbonyl carbon C(14) (206.20 ppm) was correlated with the protons H-C(10), H-C(12), and H-C(13), belonging to the HCH moiety. Taking all the NMR and MS data into account, compound **1** could be identified as 2′-*O*-acetylsalicortin, which has been previously reported in *S. pentandra* leaves [18,19]. Furthermore, Meier and Shao [29] postulated that **1** is present in the leaves and bark of *S. pentandra*; however, spectroscopic data were not included. In addition, Reichardt and Merken [30] isolated and identified **1** in *S. lasiandra* by means of NMR spectroscopy. However, comparison and careful analysis of our data highlighted that the chemical shifts of C(1), C(2), C(3), C(4), C(5), and C(6) of the phenol ring, as well as C(10) and C(11) of HCH, were wrongly annotated. The chemical shifts and their annotations obtained in this work can be found in the Supplementary Materials (Tables S1 and S2).

Furthermore, 3′-*O*-acetylsalicortin (**2**) was isolated from fraction F5-5-5 and differed from **1** by the acetylation of position C(3′) instead of C(2′) of the sugar moiety, as confirmed by the HMBC spectrum. Proton H-C(3′), resonating at 5.08 ppm, showed couplings to carbon C(2″) at 170.79 ppm. To differentiate the exact position of the acetylation, the correlation of proton H-C(2′) with carbon C(1′) was taken into consideration. For **1**, H-C(2′) had a chemical shift of 5.02 ppm indicative for the acetylation at this position, while H-C(2′) showed resonance at 3.64 ppm for **2**, confirming the absence of the acetyl group at C(2′) in compound **2**. 3′-*O*-substituted acetylsalicortin (**2**) has never been detected in *S. pentandra* before; however, it was present in twigs of *S. pseudo-lasiogyne* and *S. glandulosa* [31,32].

Moreover, 2′-*O*-acetylsalicin (**3**) was isolated from fraction F5-5-3. In comparison to the acetylated salicortin structures, salicylate **3** was lacking the HCH moiety, the absence of which can trigger the bioactivity of the compound (Figure 9), as confirmed by comparison with literature data [30,31,32]. Besides **1**, compound **3** has previously been identified in the bark of *S. pentandra* [3,20] but, also, in *S. lasiandra* leaves and twigs and *S. pseudo-lasiogyne* and *S. glandulosa* twigs [30,31,32].

Even though fraction F5-5 showed the highest anti-inflammatory potential, additional compounds were isolated from SPE fractions F6 and F7 obtained from the potent methanol extract, since these fractions indicated a slight inhibitory activity. This was of further interest in order to investigate whether the bioactivity was induced by the combined action of several compounds or by single compounds.

Thus, further salicylates, **4**, **5**, and **6**, were isolated from SPE fractions F6 and F7 (Supplementary Material Figures S6–S13). The compounds had a salicortin substructure in common, as described above for **1**. The differences between the salicylates could be shown mainly in the groups attached to the sugar moiety. While HMBC correlations of 2′,6′-*O*-diacetylsalicortin (**4**) revealed the presence of two acetyl groups bound at positions C(6′) at 64.01 ppm and C(2′) at 74.26 ppm of the sugar moiety, protons H-C(6′) of lasiandrin (**5**), resonating at 4.27 and 4.64 ppm, showed couplings to carbon C(8′) of the carboxyl group at 170.78 ppm of another HCH residue instead of an acetyl moiety. Tremulacin (**6**) comprised a benzoic acid attached to C(2′) of the sugar moiety, and the ^1^H-NMR signals of benzoic acid were shifted to a higher frequency. The protons H-C(7″) and H-C(3″), as well as H-C(4″) and H-C(6″), overlapped at 7.98 and 7.52 ppm, respectively, since they were mirrored in the phenolic ring. All three compounds have already been identified in *S. pentandra* leaves [18,19]. NMR data, however, were still missing, and structure elucidation was based on their tentative identification by HPLC/API-ES mass spectrophotometry. In accordance with our work, Kim et al. [31] and Yang et al. [32] could identify the compounds in twigs of *S. pseudo-lasiogyne* and *S. glandulosa* by means of NMR spectroscopy.

Further fractionation of fraction F5-2-2 by means of HPLC revealed a pseudo molecular ion of *m*/*z* 487, indicating a molecular formula of [M+HCO_2_H-H]^-^ for cinnamyl glycoside cinnamrutinose A (**7**) (Supplementary Material, Figures S14 and S15). In comparison to the described salicylates in the current work, the double bond of **7** was *trans*-configured, as shown by the coupling constants of ^3^*J*_H-H_ = 16.14 Hz at position H-C(2). A slight impurity of the compound showed a pseudo molecular ion of *m*/*z* 489 [M+HCO_2_H-H]^-^ and indicated the absence of the double bond. This observation was confirmed by the ^1^H-NMR and ^13^C-NMR spectra of the impurity, with protons H-C(3) and H-C(2) of both methylene groups resonating at 2.7 and 1.9 ppm and the carbons at 31.74 and 31.37 ppm, respectively. However, a complete structure elucidation of the slight impurity was not possible, since the signals were very weak. The two sugars, *L*-rhamnose and *D*-glucose, as part of the compound were determined by a derivatization reaction and subsequent MS^2^ screening (method adopted from Schmid et al. [33]). Based on the HMBC experiment of **7**, the proton H-C(6′) of *D*-glucose, resonating at 3.88 ppm, is coupled with carbon C(1″) of *L*-rhamnose at 101.66 ppm; thus, the two sugars are bound to each other. Characteristic in the same HMBC experiment was also the coupling of the methylene protons of the cinnamoyl-group H_α_-C(1) and H_β_-C(1) at 4.24 and 4.43 ppm, respectively, with C(1′) of *D*-glucose at position 102.85 ppm. In particular, the NMR experiments (Supplementary Material, Table S4) could confirm the binding of the two sugars, as well as the binding of *D*-glucose to the cinnamoyl group and, thus, the structure of cinnamrutinose A (**7**). Compound **7** has already been described in the stems of *S. triandra* × *dasyclados* and *Populus tremula* and *P. euphratica* leaves, belonging to the *Salicaceae* family [32,34,35,36]. However, until now, compound **7** has not been identified in *S. pentandra*, and data on its bioactivity are still missing.

As described above, all structures of the isolated compounds were confirmed by mass spectrometry and 1D/2D NMR experiments, but for the overall structure elucidation, the absolute configurations of **1**, **2**, **4**, **5**, and **6** were examined by CD (circular dichroism) spectroscopy, showing negative ellipticity values (see the Supplementary Materials Figure S16). The molar ellipticity (Δε) values at the chiral carbon C(9) detected at 78.81 ppm of **1**, **2**, **4**, **5**, and **6** were compared with the literature investigating salicylates from *P. trichocarpa* x *deltoides* leaves and *S. pseudo-lasiogyne* twigs [32,37]. Similar molar ellipticity values were found for **1** (Δε = −15.5 mdeg), **2** (Δε = −11.7 mdeg), **4** (Δε = −14.4 mdeg), and **5** (Δε = −17.2 mdeg). The absolute configuration of **5** has never been examined before and is determined in the present work as an *S*-configuration. For **6**, two negative ellipticity values and two wavelength maxima were determined: Δε = −19.6 and −8.8 mdeg (*c* 0.38 mM, CH_3_OH, λ_max_ = 228 and 209 nm). However, Δε = −19.6 mdeg showed a lower ellipticity value than the postulated value by Feistel et al. [37] as −10.5 mdeg, and the wavelength was 239 nm. The different values may be due to changed temperatures during the measurements [38], even though the same solvent (methanol) was used. Based on these data, all the compounds isolated from *S. pentandra* held an absolute *S*-configuration (see the Supplementary Materials Figure S16), as described in the literature [32,37]. These findings correlate very well with one part of the biosynthetic pathway of salicylates, the shikimate pathway. The synthesis of salicylates begins with *L*-phenylalanine [39] and, thus, may explain the *S*-configuration at the chiral center.

Besides fractions F5-2, F5-5, F6-12, F6-13, and F7-8, fraction F7-4 was purified by means of semipreparative HPLC in order to isolate a compound with a similar pseudo molecular ion as **1** and **2**. The compound of F7-4 was initially supposed to be an acetylsalicortin isomer. Subfraction F7-4-6 (see the Supplementary Materials Figure S17) was collected for structure elucidation, removed from the organic solvent, and lyophilized. After reinjection into the HPLC system, the peak of fraction F7-4-1 had a higher intensity than the peaks of fraction F7-4-6 (see the Supplementary Materials Figure S17), which initially indicated either degradation or a chemical reaction, such as isomerization of the compounds.

**Figure 5:**
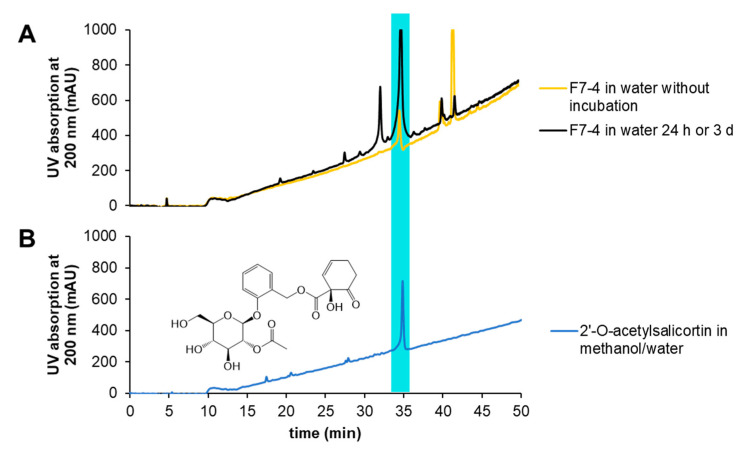
Analytical HPLC chromatograms of fraction F7-4 diluted in water before ((**A**), yellow) and after ((**A**), black) incubation in comparison to 2′-*O*-acetylsalicortin (**1**) ((**B**), turquoise).

In order to gain further knowledge about the behavior of this fraction and possible degradation or isomerization, subfractions of F7-4 were stored at room temperature for 24 h or under cell culture conditions in water at 37 °C for 24 h with and without exposure to light. After storage, the fraction showed chromatographic shifts in the retention time. Using analytical HPLC on a PFP (pentafluorophenyl) column, the retention time shift before and after storage was proven (Figure 5). Indeed, the intensity of the peak at about 40 min (Figure 5A) was reduced over time, and it was supposed that 2′-*O*-acetylsalicortin, eluting at approximately 35 min, was produced (Figure 5A,B), as the peaks F7-4-1 and F7-4-6 showed the same pseudo molecular ion of *m*/*z* 465, which was also detected for acetylsalicortin, by means of LC-ToF-MS. This was approved by the co-chromatography of pure 2′-*O*-acetylsalicortin (Figure 5B, turquoise).

**Figure 6:**
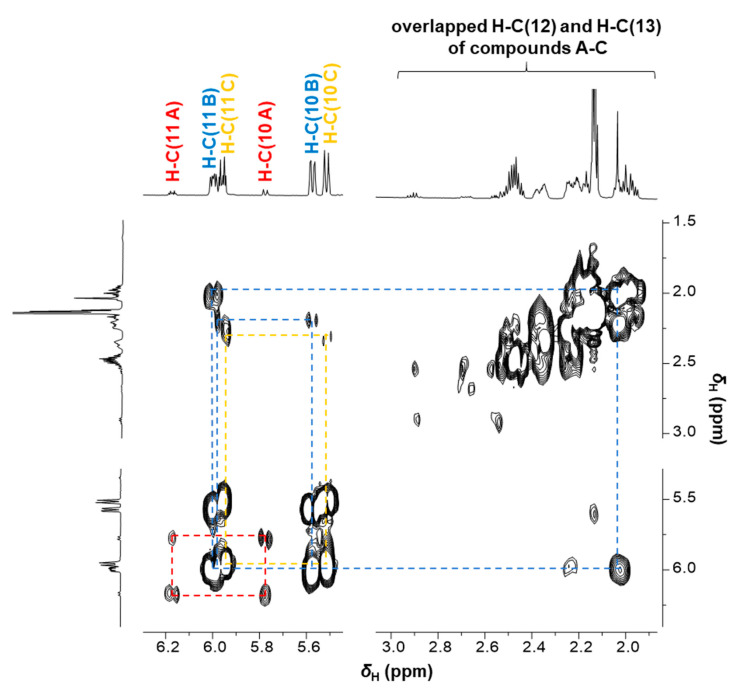
Excerpt of the COSY spectrum of fraction F7-4 indicating three compounds: A (red), B (blue), and C (yellow). The two-dimensional NMR spectrum shows the correlations of the protons H-C(10), H-C(11), H-C(12), and H-C(13).

In order to elucidate the structure of the compounds present in fraction F7-4 before incubation (Figure 5A), further experiments were performed. First, the fraction was analyzed by two-dimensional NMR (Supplementary Material Tables S5 and S6), showing three different compounds (A-C) in the COSY spectrum between 5.5 and 6.2 ppm (Figure 6) and differing in the chemical shifts. In the HSQC experiment, the carbon signals at 104.47 and 111.81 ppm did not show a correlation to any proton, indicating quaternary carbon atoms C(14 B) and C(14 C) (data not shown).

**Figure 7:**
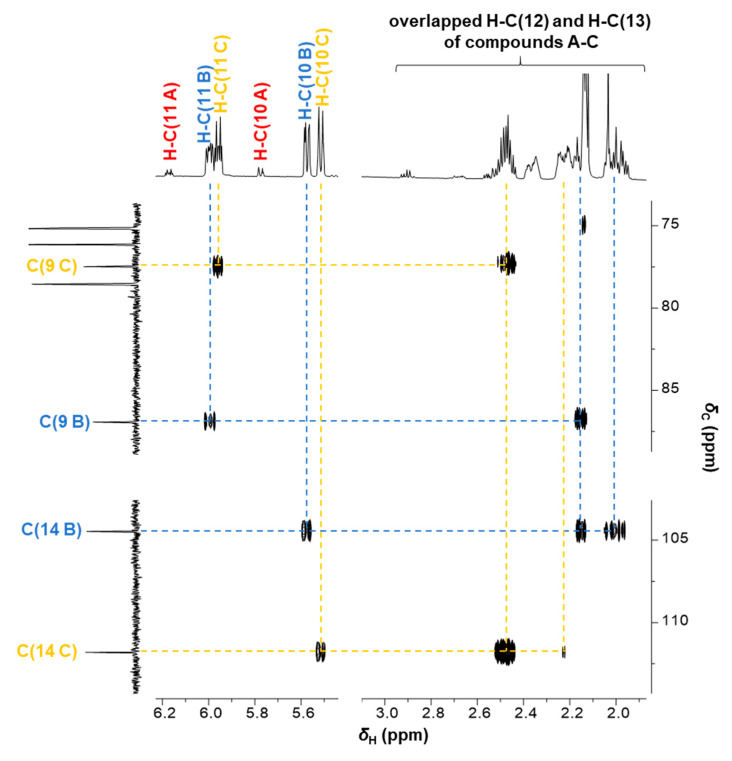
Excerpt of the heteronuclear multiple bond correlation (HMBC) experiment of fraction F7-4, indicating the correlations of the protons H-C(10) and H-C(13*α* and 13*β*) with the carbon C(14) of the compounds B (blue) and C (yellow), which are shifted to a lower frequency in comparison to carbon C(14 A), as part of the 2′-*O*-acetylsalicortin structure, and resonating at 207.4 ppm (not shown).

In the HMBC spectrum, couplings of C(9) with H-C(11) and C(14) with H-C(12) and H-C(13) of the two compounds B and C of fraction F7-4 were detected, confirming the HCH moiety (Figure 7). Based on the observations in the HMBC spectrum and the chemical shift of C(14) at 104.47 (compound B) and 111.81 ppm (compound C), respectively, it was hypothesized that position C(14) consists of geminal diols, since no proton could be assigned. The proposed pseudo molecular ion of the diastereomeric compounds B and C containing a diol with *m*/*z* 483 ([M-H]^−^) was not detected by LC-ToF-MS analysis. Therefore, in-source fragmentation (water loss) under the given mass spectrometric conditions was assumed to reveal the fragment ions of *m*/*z* 465 ([M-H_2_O-H]^-^) and not the expected pseudo molecular ions (*m*/*z* 483).

In order to prevent in-source fragmentation and to confirm the diol structure, the hydroxy groups at position C(14) were protected by an acetalization reaction. The *m*/*z*-value of 563.21, obtained by LC-ToF-MS analysis after acetalization, indicated the binding of a second acetal group at the alcohols of the sugar moiety at positions C(3′) and C(4′).

**Figure 8:**
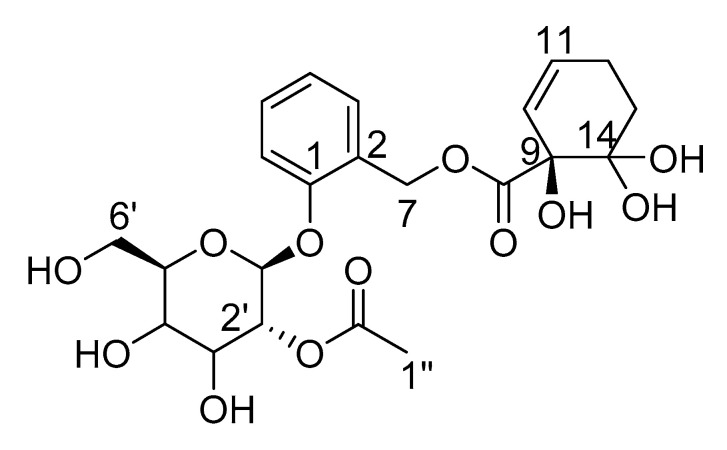
*β*-d-Glucopyranoside, 2-[[[(1-hydroxy-6,6-dihydroxy-2-cyclohexen-1-yl)dihydroxy]oxy]methyl]phenyl, 2-acetate comprising a geminal diol at position C(14) of the HCH moiety.

Indeed, the two diastereomeric compounds comprising two hydroxyl groups at position C(14) are oxidized to 2′-*O*-acetylsalicortin during incubation, and the structure of *β*-d-glucopyranoside, 2-[[[(1-hydroxy-6,6-dihydroxy-2-cyclohexen-1-yl)dihydroxy]oxy]methyl]phenyl, 2-acetate (**8**) was elucidated (Figure 8). However, an exact assignment of the protons of both diastereomers B and C holding a geminal diol was not possible, since the protons and, especially, H-C(12) and H-C(13) were overlapping in the NMR spectrum (Figure 6 and Figure 7). Nevertheless, all carbon atoms could be assigned to the three compounds. Moreover, a comparison of the NMR data with purified reference compound **1** confirmed compound A to be 2′-*O*-acetylsalicortin. Thus, the first insights into the chemical composition of fraction F7-4 could be obtained; however, for single compound isolation, more purification steps need to be done using derivatization reagents prior to fractionation in order to protect the diol group. In the future, the yield of the derivatized fraction needs to be increased in order to analyze the bioactivity of both diastereomers B and C.

### 2.2. Anti-Inflammatory Activity of Salix Compounds

**Figure 9:**
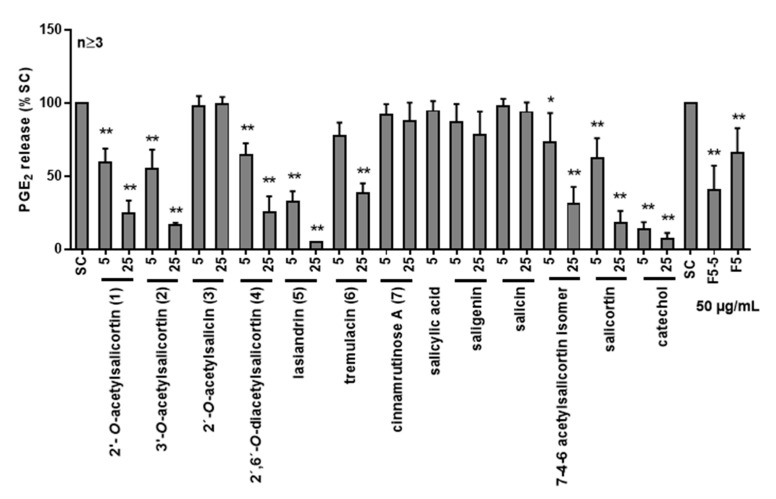
Anti-inflammatory activity of the isolated compounds **1**–**7** and new diastereomeric compound of F7-4-6 from *S. pentandra* methanol extract and the possible degradation/metabolization compounds salicylic acid, saligenin, salicin, salicortin, and catechol. PGE_2_ release was quantified in LPS-stimulated PBMC from healthy donors. Cells were stimulated with LPS for 24 h after pretreatment with the isolated compounds for 30 min. Data are the means + SD of at least four independent experiments expressed as percentages of the solvent control. Asterisks indicate statistically significant differences between the respective treatment and the solvent control. * *p* < 0.05 and ** *p* < 0.01. SC = solvent control (1% dd water).

Isolated compounds **1**–**7** from SPE fractions F5, F6, and F7 and **8** from fraction F7-4-6, as well as commercially obtained substances, including salicylic acid, salicin, salicortin, and catechol, were tested for their anti-inflammatory potential (5 and 25 µg/mL). The selected, commercially available compounds were reported as degradation or metabolization products of salicylates [19,40] and were included to investigate whether the compounds themselves or their degradation or metabolization products induce an anti-inflammatory effect. The compound HCH could not be obtained commercially. Salicylic acid, salicin, **3**, and **7** did not block the PGE_2_ release in LPS-stimulated PBMC (Figure 9). Compound **6** showed an inhibition only at 25 µg/mL. Fraction F7-4-6 comprising two diastereomers, salicortin, catechol, **1**, **2**, **4**, and **5** inhibited the PGE_2_ release at both concentrations, whereas catechol had the highest efficacy of all the tested compounds at 5 µg/mL. Salicortin and **6** have already been identified in an ethanolic extract from the *Salix* cortex as anti-inflammatory substances using the ICAM-1 (intercellular adhesion molecule-1) assay in TNF-α-induced endothelial cell cultures [12,41]. Then, 50 μM salicortin reduced the ICAM-1 expression to 52.4% and tremulacin in the same concentration to 75.0% of the control [12,41]. In our study, salicortin also exhibited higher anti-inflammatory potential than tremulacin. At a comparable concentration, 25 µg/mL salicortin (=58.82 µM) inhibited the PGE_2_ release from PBMC to 28% and tremulacin (=47.30 µM) to 37% of the LPS-stimulated cells. In addition, it was postulated that salicortin comprising a HCH moiety degraded to salicylic acid and catechol under cell culture conditions [12], as well as alkaline conditions [19]. Hence, Knuth et al. [12] and Knuth [41] attributed the reduction in TNFα-induced ICAM-1 expression to the degradation of salicortin and tremulacin to catechol. Freischmidt et al. [42] revealed catechol as one of the bioactive compounds in an aqueous willow bark extract prepared from *S. purpurea* L. (STW 33-I) that also decreased TNF-α-induced ICAM-1 expression by liquid/liquid extraction [42]. Thus, the presence and metabolism of catechol seemed to be one determinant of the anti-inflammatory potency of willow bark extract [42].

Moreover, comparing the acetylsalicortin compounds with regards to their nitric oxide inhibitory potential, 3′-*O*-acetylated salicortin (**2**) exhibited higher potency in LPS-stimulated murine microglia BV2 cells than 2′-*O*-acetylated salicortin (**1**), indicating an impact of the acetylation position on the bioactivity [31]. The results of our study on PGE_2_ release in LPS-stimulated PBMC did not show a similar potency, since **1** (5 µg/mL 59%+/−9% and 25 µg/mL 25%+/−8%) and **2** (5 µg/mL 55%+/-13% and 25 µg/mL 16%+/−2%) revealed similar bioactivity. Compound **4** (5 µg/mL 64%+/−8% and 25 µg/mL 25%+/−11%), comprised of two acetyl-groups, also revealed a similar anti-inflammatory potential in PGE_2_ inhibition.

On the other hand, 2′-*O*-acetylsalicin (**3**), composed of saligenin and 2′-*O*-acetylated glucose, showed a very low in vitro PGE_2_ inhibitory potential compared to the other isolated salicylates (**1**, **2**, **4**, **5**, and **6**) comprising the salicortin substructure, as shown in Figure 9. Thus, the absence of the HCH moiety may reduce the anti-inflammatory effect of compound **3**. A higher efficiency against PGE_2_ release was found for lasiandrin (**5**; 5 µg/mL 33%+/−7% and 25 µg/mL 5%+/−0%), comprising two HCH moieties and an acetylated glucose (Figure 9). The effect was comparable to catechol (5 µg/mL 14%+/−5% and 25 µg/mL 8%+/−4%) at 25 µg/mL, showing that salicylate **5** was one of the most bioactive among the isolated compounds. Previous studies have already demonstrated the metabolization mechanism of HCH. The moiety degrades to catechol, which showed a high efficacy in our study [19]. For **5**, the two HCH moieties may mediate the anti-inflammatory potential, which could be explained by the metabolization pathway.

Among the isolated salicylates, **6**, comprising a benzoic acid attached to glucose, had a lower inhibitory activity against PGE_2_, and thus, the attached benzoic acid could lead to a reduced activity of the compound. Kim et al. [31] postulated a neuroprotective effect of a similar salicortin derivative; however, the benzoyl group was attached to position C(3′). As mentioned previously, acetylation or benzoylation at position C(3′) may enhance the neuroprotective activity [31]. However, 3′-*O*-benzoylated salicortin was not investigated in this study, since it was not isolated by application of the activity-guided fractionation approach. Thus, no conclusion about its enhancing effect on PGE_2_ inhibition, compared to 2′-*O*-benzoylated salicortin (**6**), could be drawn.

## 3. Conclusions

In this study, in vitro immune responses of human peripheral blood mononuclear cell spectroscopic and spectrometric experiments were combined to provide evidence about the anti-inflammatory potential of isolated and newly identified compounds from the bioactive methanol extract of *S. pentandra*. Following the activity-guided fractionation approach, seven compounds could be isolated and structurally characterized. As compared with the recent literature, the HCH and acetyl moiety may enhance the anti-inflammatory activity of the isolated salicylates. In turn, the absence of the HCH group of salicylate compounds may prevent PGE_2_ inhibition, as shown for **3**. Between the seven isolated compounds, **5** showed the highest anti-inflammatory potential. Since it was extracted from a less potent SPE fraction, the high bioactivity could be explained due to the two attached HCH moieties that are unique between the isolated compounds and may degrade to catechol, a highly bioactive compound. Moreover, fraction F5-5 containing three salicylates showed a higher anti-inflammatory potential than fraction F6-13, from which **5** was isolated. Thus, it can be assumed that the composition of F5-5, but not the single compound **3** contained in the fraction, is bioactive. The non-salicylate, **7**, was found for the first time in the species *S. pentandra*; nevertheless, it did not show any anti-inflammatory effect. Moreover, we discovered two new diastereomeric compounds: *β*-d-glucopyranoside and 2-[[[(1-hydroxy-6,6-dihydroxy-2-cyclohexen-1-yldihydroxy]oxy]methyl]phenyl, 2-acetate in fraction F7-4 consisting of a geminal diol at the HCH moiety, which also provided evidence of the anti-inflammatory potential in a mixture with **1**. Nevertheless, further experiments are needed for the proper structure elucidation of both diastereomers.

Even though polyphenols have been discussed as potentially important for the therapeutic effect of willow bark [9,43], our activity-guided fractionation experiments, as well as the present in vitro data on PGE_2_ inhibition, do not support this. Our data show great differences in the anti-inflammatory activity between the identified salicylates and raise questions about the adequacy of the current pharmaceutical extract standardization procedures. This finding should thus be investigated further to clarify the relevance of the in vitro observations for the overall therapeutic efficacy. The breeding of new *Salix* species towards high concentrations of the most bioactive compounds identified in this study should also be considered.

## 4. Materials and Methods

### 4.1. Chemicals

The following chemicals were obtained commercially: acetonitrile and methanol (HPLC gradient grade and LC-MS grade reagent; J.T. Baker, Neventer, The Netherlands); formic acid (Merck, Darmstadt, Germany); ethyl acetate (BDH, Prolabo, Briare, France); hydrochloric acid (fuming, 37%; Merck KGaA, Darmstadt, Germany); *L*-cysteine methyl ester hydrochloride (purity 98%; Sigma-Aldrich, Darmstadt, Germany); anhydrous pyridine (purity 99.8%; Sigma-Aldrich, Darmstadt, Germany); phenylethyl isothiocyanate (Sigma-Aldrich, Steinheim, Germany); the monosaccharides *D*-glucose, *L*-glucose, and *L*-rhamnose (Sigma-Aldrich, Darmstadt, Germany); *L*-galacturonic acid (Serva Feinbiochemie, Heidelberg, Germany); and *D*-glucuronic acid (Fluka Chemika, Buchs, Switzerland). The compounds *D*-salicin (purity ≥ 98%; Carbosynth, Bertshire, UK), salicylic acid (purity ≥ 99%; Carl Roth GmbH & Co. KG, Karlsruhe, Germany), acetyl salicylic acid (purity ≥ 99%; Sigma-Aldrich, Darmstadt, Germany), saligenin (purity ≥ 99%; Sigma-Aldrich, Darmstadt, Germany), and catechol (purity ≥ 95.0% (GC); Sigma-Aldrich, Darmstadt, Germany) were commercially obtained, and (*S*)-salicortin (purity 95%) was purified and isolated by Biosynth Carbosynth (Compton, UK) from *Populus* sp. (*Poplar*). The following deuterated chemicals supplied from Sigma-Aldrich (Darmstadt, Germany) were used for the NMR experiments: DMSO-*d_6_*, acetone-*d_6_*, methanol-*d_4_*, and acetonitrile-*d_3_*. Ultrapure water for the chromatographic separation was taken from the water purification systems Milli-Q^®^ Advantage A10 (Millipore, Schwalbach, Germany) and Elix^®^ (Merck S.A.S., Molsheim, France).

Fetal bovine serum (FBS) South American, *L*-glutamine solution, RPMI-1640, PBS buffer (without Ca^2+^ and Mg^2+^), and penicillin/streptomycin solution (10,000 U/mL and 10,000 µg/mL) were supplied from Gibco™, Life Technologies GmbH (Darmstadt, Germany). Lipopolysaccharide (LPS; from *Escherichia coli* O11:B4) and ethanol absolute were obtained from Sigma Aldrich (Taufkirchen, Germany). LymphoPrep^TM^ gradient was purchased from PROGEN Biotechnik GmbH (Heidelberg, Germany). ROTISOLV^®^ HPLC gradient grade water and dimethyl sulfoxide (DMSO; purity >99%) were acquired from Carl Roth (Karlsruhe, Germany) and Applichem GmbH (Darmstadt, Germany), respectively.

### 4.2. Sequential Solvent Extraction of Willow Bark

The *Salix* spp. plants were cultivated and harvested as described in Förster et al. [44]. Raw ground willow bark powder (120 g) from branches of the woody plant *S. pentandra* was obtained from Humboldt-Universität zu Berlin, Germany. Then, the willow bark powder was extracted with 680 mL methanolic solvent by stirring at room temperature for 30 min. After vacuum filtration, the residue was collected and re-extracted another four times with methanol, yielding the methanol extract. Then, the residue was used for the extraction with three times methanol/water (*v*/*v*, 70/30; methanol/water extract) and, finally, with three times water (water extract) using the same conditions. The organic solvent of each of the three phases was removed under reduced pressure using a rotary evaporator, and the residual aqueous layers were freeze-dried. The methanol, methanol/water, and water extracts were stored at −20 °C until further use.

### 4.3. Fractionation of the Methanol Fraction by Means of Solid-Phase Extraction

The bioactive methanol extract was fractionated by solid-phase extraction into eleven fractions (10% steps). Therefore, the lyophilized extract was dissolved in water and sonicated in an ultrasonic bath for 10 min. First, the material of the C_18_ end-capped cartridges (60 Å, 10 g/70 mL packing volume; CHROMABOND^®^, Macherey-Nagel GmbH & Co. KG, Düren, Germany) was conditioned with methanol (70 mL) and methanol/water (*v*/*v*, 70/30; 70 mL) and, finally, equilibrated with water. Then, the methanol extract, dissolved in water, was added onto the cartridge obtaining the first fraction (F1) through vacuum filtration. F2 was eluted using methanol/water (*v*/*v*, 10/90). The following fractions were attained by using increasing amounts of methanol (10% elution steps) until the final step, yielding fraction F11 by using a pure methanolic solvent. The collected fractions were evaporated under reduced pressure, lyophilized, and stored at −20 °C until further use.

### 4.4. Isolation and Identification of (Non)Bioactive Salix Compounds

SPE fractions F5, F6, and F7 were dissolved in methanol, filtered by membrane filters (Minisart^®^ RC 15, pore size 0.45 µm, Ø 15 mm; Sartorius AG, Göttingen, Germany), and separated chromatographically by preparative HPLC into six (F5-1 to F5-6) and fourteen (F6-1 to F6-14 and F7-1 to F7-14) subfractions, according to the UV signals at 200 nm. Then, the organic solvent of each subfraction was removed by rotary evaporation, freeze-dried, and used further to assess the bioactivity. The purity and complexity of each fraction was examined by analytical HPLC-UV (200 nm), LC-ToF-MS, and NMR spectroscopy. A subsequent semipreparative HPLC gradient development allowed the isolation of individual compounds (see the Supplementary Materials for details). Consequently, the structure determination was performed by means of LC-MS, LC-ToF-MS, 1D/2D-NMR, and CD spectroscopy.

The chromatographic and spectroscopic conditions and parameters are appointed in the Supplementary Materials. 2′-*O*-Acetylsalicortin (**1**), 3′-*O*-acetylsalicortin (**2**), 2′-*O*-acetylsalicin (**3**), and cinnamrutinose A (**7**) were isolated from fraction F5; 2′,6′-*O*-diacetylsalicortin (**4**) and lasiandrin (**5**) from SPE fraction F6; and tremulacin (**6**) from fraction F7 (Figure 3).

### 4.5. Determination of the Absolute Configuration

Circular dichroism spectroscopy was performed to determine the absolute configuration of the salicylates **1**, **2**, **4**, **5**, and **6** comprising a HCH moiety and, thus, a chiral carbon C(9). Therefore, the measurements were performed on the J-810 spectropolarimeter (Fern-UV CD Spektrum; Jasco, Pfungstadt, Germany) operated with the PT-423S Peltier element, kindly provided by the Chair of Biological Chemistry (TU Munich, Head: Prof. Dr. Arne Skerra). Throughout the experiments, the temperature was 20 °C, and the manually controlled nitrogen gas (N_2_) was kept between 3 and 4 L. Each compound was dissolved in methanol (0.2 mg/mL), transferred into a quartz cuvette with a pathlength of 1 mm, sealed with a cap to prevent solvent evaporation, and scanned eight times over a wavelength of 185–350 nm. A spectral analysis showing the ellipticity (mdeg) and wavelength was performed with Spectra Manager^TM^ software (version 1.17.00; Jasco, Tokyo, Japan).

### 4.6. Sugar Determination

The sugar moieties of the isolated and purified (non)salicylates **1**–**7** were determined, employing the protocol adopted from Schmid et al. [33]. Fifty microliters of deuterated NMR solvent containing pure compounds (about 8.33 mg/mL) were evaporated under nitrogen gas. The compounds were hydrolyzed under acidic conditions using 500 µL of 2 M HCl and shaking for 1 h at 1400 rpm and 100 °C to cleave the sugar moiety from the rest of the compound. After removing the solvents, the dry compounds were diluted in 750 µL of water, and extraction of the sugar moiety was performed twice using ethyl acetate (750 µL). The water layers of each compound containing the sugars were dried using the speedVac vacuum concentrator plus with an integrated membrane vacuum pump (without rotor, 230 V/50–60 Hz; Eppendorf, AG, Hamburg, Germany). The extracted sugar residues and 1 mg of each reference compound (*D*-glucose, *L*-glucose, *D*-galactose, *D*-galacturonic acid, *D*-glucuronic acid, and *L*-rhamnose) were diluted in 1 mL of *L*-cysteine methyl ester hydrochloride in anhydrous pyridine (2 mg/mL). Derivatization was performed by shaking the samples for 1 h at 1400 rpm and 60 °C, followed by adding 5 µL of phenylethyl isothiocyanate and shaking using the same conditions. Consequently, the derivatized monosaccharide samples were dried using the speedVac vacuum concentrator and diluted in 500 µL acetonitrile/water (*v*/*v*, 1/1) for screening by means of QTRAP-LC-MS (details in the Supplementary Materials). The retention times of the reference compounds were compared with the derivatized sugars of the isolated compounds **1**–**7**.

### 4.7. Acetalization Reaction

For the determination of the possible geminal diol structures in fraction F7-4, an acetalization reaction was performed. Therefore, 0.05 mg of the catalyst *p*-toluenesulfonic acid was added to 3.6 mg of fraction F7-4. Subsequently, 300 µL of anhydrous acetone was added to the sample. Next, the sample was incubated for 24 h at room temperature to allow acetalization of the diols. The acid was neutralized by adding an equal amount of NaHCO_3_, and the solvent was evaporated and, finally, diluted in 300 µL methanol/water (*v*/*v*, 70/30). The precipitated sample was centrifuged, and the supernatant was analyzed by LC-ToF-MS.

### 4.8. High-Performance Liquid Chromatography

The HPLC devices (Jasco, Gross-Umstadt, Germany) consisted of the following equipment: binary high-pressure HPLC pump system (PU-2080 Plus), autosampler (AS-2055), degasser (DG-2080-53; 3-line), DAD (diode array detection) detector (MD-2010 Plus) or UV/VIS detector (UV-2075), and injection valve (Rh 7725i type Rheodyne; Bensheim, Germany). Chromatographic gradient development and peak separation was performed on an analytical 250 × 4.6 mm, 5 µm Luna^®^ phenyl-hexyl column (Phenomenex Ltd., Aschaffenburg, Deutschland) or Luna^®^ pentafluorophenyl column (Phenomenex Ltd., Aschaffenburg, Deutschland). Furthermore, (sub)fractionation was carried out through chromatographic separation on a preparative 250 × 21.2 mm Luna^®^ phenyl-hexyl column with a particle size of 5 µm (Phenomenex Ltd., Aschaffenburg, Deutschland) and a semipreparative 250 × 10 mm, 5 µm Luna^®^ PFP (Phenomenex Ltd., Aschaffenburg, Deutschland) or a semipreparative 250 × 10 mm, 5 µm Luna^®^ phenyl-hexyl column (Phenomenex Ltd., Aschaffenburg, Germany). Each column was attached to a suitable precolumn. The analyzed data was processed by ChromPass (version 1.9; Jasco Groß-Umstadt, Germany) or Galaxie software (version 1.10; Agilent Technologies, Oberhaching, Germany).

### 4.9. Ultra-Performance Liquid Chromatography Time-of-Flight Mass Spectrometry

The isolated compounds **1**-**7** were analyzed by means of the Synapt G2 HDMS UPLC-ToF-MS system (Waters UK Ltd., Manchester, UK) coupled to an Acquity UPLC core system (Waters) recording high-resolution mass spectra. Chromatographic separation was performed on a BEH C18 column (150 × 2.1 mm, 1.7 µm, Waters) with a flow rate of 0.4 mL/min. The injection volume was set to 1 µL, and the chromatography was run for 8 min, employing 0.1% formic acid in water (solvent A) and 0.1% formic acid in acetonitrile (solvent B) and starting the chromatography with a mixture of 1% B for 1 min and then increasing eluent B to 60% within 3.5 min, to 80% B in 1 min, to 100% B in 1.5 min, held isocratic for another 1 min, decreased again to 1% B in 0.5 min, and finally, held at 1% B for 1.5 min. Data analysis and processing was performed by MassLynx software (version 4.1; Waters).

### 4.10. Quadrupole LC-MS/MS Spectrometer

In order to obtain further structural information, the fragmentation pattern of the isolated compounds **1**–**7** was analyzed by means of a flow injection analysis. The MS^2^ spectra of each compound were acquired in the negative ionization mode on a QTRAP 6500 LC-MS/MS system (Sciex, Darmstadt, Germany) after optimizing the declustering potential and the collision energy for each compound.

### 4.11. Nuclear Magnetic Resonance Spectroscopy (NMR)

Structure determination of all the isolated compounds was performed by one- and two-dimensional NMR experiments. The following two devices were used: the Bruker UltraShield^TM^ Plus AVANCE III 500 MHz and UltraShield^TM^ Plus 9.4 T magnet AVANCE Neo 600 MHz spectrometers (Bruker, Rheinstetten, Germany), both equipped with a 300-K Triple Resonance Cryo-TCI probe (Bruker). A quantitative NMR (qHNMR) analysis was conducted using the Bruker AVANCE III 400-MHz system (Bruker) with a Z-gradient 5-mm multinuclear observe probe (BBFO_PLUS_; Bruker) through signal integration and external calibration of the spectrometer with the ERETIC 2 tool using the PULCON method as reported earlier [45]. For the external calibration, the standard *L*-tyrosine was used. The concentration of each analyte was determined according to the following formula [45]:

$$C_{U}=kC_{\mathrm{ref}}\frac{A_{U}T_{U}\theta_{90}^{U}n_{\mathrm{ref}}}{A_{\mathrm{ref}}T_{\mathrm{ref}}\theta_{90}^{\mathrm{ref}}n_{U}}$$

Acetonitrile-*d_3_* (*δ*_H_ = 1.94 ppm) and methanol-*d_4_* (*δ*_H_ = 3.31 ppm) were used as the solvents, and the chemical shifts are reported in parts per million relative to the solvent signals. The following qHNMR signals were used for integration: 7.16 ppm (H-C(6), 1H) and 5.74 ppm (H-C(10), 1H) for **1**, 7.17 ppm (H-C(6), 1H) and 7.07 ppm (H-C(4), 1H) for **2**, 7.39 ppm (H-C(3), 1H) and 7.14 ppm (H-C(6), 1H) for **3**, 7.32 ppm (H-C(3), H-C(5), 2H) and 7.11 ppm (H-C(4), H-C(6), 2H) for **4**, 5.73 ppm (H-C(10), H-C(10′), 2H) and 6.14 ppm (H-C(11), H-C(11′), 2H) for **5**, 7.64 ppm (H-C(5′′), 1H) and 7.04 ppm (H-C(4), 1H) for **6**, 6.35 ppm (H-C(2), 1H) and 6.69 ppm (H-C(3), 1H) for **7**, 6.81 ppm (H-C(3), H-C(6), 2H) and 6.72 ppm (H-C(4), H-C(5), 2H) for catechol, 7.05 ppm (H-C(4), 1H) and 7.35 ppm (H-C(5), 1H) for salicin, 7.85 ppm (H-C(3), 1H) and 7.52 ppm (H-C(5), 1H) for salicylic acid, 7.37 ppm (H-C(4), 1H) and 7.98 ppm (H-C(3), 1H) for acetyl salicylic acid, 7.11–7.18 ppm (H-C(3), H-C(5), 2H) and 6.80–6.86 ppm (H-C(4), H-C(6), 2H) for saligenin, and 7.22 ppm (H-C(6), 1H) and 7.04 ppm (H-C(4), 1H) for salicortin. The integrals of the signals of the compounds were compared with integrated signals not belonging to the compound to determine the purity. Moreover, the theoretical concentration was compared to the calculated one for additional purity determination. All compounds isolated from *S. pentandra* had a purity ≥ 95%, except **3** and **7**. Salicortin, which was obtained commercially, showed a purity of 92%. The data was processed and evaluated by means of TopSpin^TM^ 3.6.0 (Bruker, Rheinstetten, Germany) and MestReNova 12.0.3 (Mestrelab Research S.L., Santiago de Compostela, Spain).

### 4.12. Isolation and Exposure of Human Peripheral Blood Mononuclear Cells

The study was approved by the Ethics Committee of the University of Freiburg, Germany and was carried out according to the guidelines of the Declaration of Helsinki. Human PBMC were isolated from buffy coats of healthy adult donors, which were received from the blood transfusion center at the University Medical Center Freiburg, Germany, as described before [22]. Isolated PBMC were diluted in RPMI 1640 medium supplemented with 10% heat-inactivated FBS, 2 mM *L*-glutamine, 100 U/mL penicillin, and 100 µg/mL streptomycin, pretreated with *Salix* cortex extracts, fractions, the compounds **1**–**7**, salicin, salicylic acid, salicortin, saligenin, and acetyl salicylic acid or the solvent for 30 min and subsequently stimulated with 100 ng/mL LPS at 37 °C in a humidified incubator with 5% CO_2_/95% air atmosphere for 24 h.

### 4.13. Preparation of Extracts, Fractions, and Compounds for Bioactivity Assays

The methanol, methanol/water, and water extracts, eleven SPE fractions, isolated compounds, and commercially obtained compounds were dissolved in distilled water to determine the anti-inflammatory potential activity. Therefore, 10 mg/mL of each of the three extracts and 5 mg/mL of the SPE fractions derived from the methanol extract were dissolved in water, relating to the natural concentrations (based on the methanol extract), whereas the subfractions F5-1 to F5-6 were diluted in DMSO. Fraction F7-4-6 and the single compounds were diluted in distilled water and stored at −80 °C until further use.

### 4.14. Quantification of PGE_2_ Release by Enzyme-Linked Immunosorbent Assay Assay

To quantify the PGE_2_ release, cell-free supernatants were used for the photometric quantification of PGE_2_ using ELISA (PGE_2_ ELISA kit, Cayman Chemical, Hamburg, Germany) according to the manufacturer’s instructions.

### 4.15. Determination of COX-1 and COX-2 Enzyme Activity Inhibition

The inhibitory effect of the extracts and SPE fractions on the COX enzyme activity was determined using Cayman COX (human) Inhibitor Screening Assay kits according to the protocols of the manufacturer (Cayman, Hamburg, Germany) and as described previously [28]. Briefly, the human recombinant COX-1 or COX-2 enzyme was incubated with extracts or fractions for 8 min at 37 °C. Subsequently, the reaction was initiated by adding arachidonic acid and incubating the mixture for 30 s at 37 °C. Enzyme catalysis was stopped by adding a saturated stannous chloride solution. Then, PGF2α release was quantified using ELISA. Acetyl salicylic acid (ASA) was used as the positive control.

### 4.16. Statistics

The bioassay data were processed by GraphPad Prism 6.0 software (La Jolla, CA, USA) and were presented as the mean + standard deviation (SD). Statistical significance was analyzed by means of the ordinary one-way ANOVA test, followed by Dunnett’s multiple comparison test. *p* values < 0.05 (*) were considered statistically significant and <0.01 (**) were considered highly statistically significant.

## Data Availability

Not applicable.

## Supplementary Materials

The following are available online at https://www.mdpi.com/article/10.3390/ijms222011138/s1: Figure S1: Preparative UV-HPLC chromatogram of SPE fraction F5 at 200 nm. Figure S2: Semi-preparative UV-HPLC chromatogram of fraction F5-5 at 200 nm. Figure S3: Centroided MS^2^ spectrum of 464.9 Da showing the fragmentation pattern of 2’-*O*-acetylsalicortin (**1**). Figure S4: Centroided MS^2^ spectrum of 465.0 Da showing the fragmentation pattern of 3’-*O*-acetylsalicortin (**2**). Figure S5: Centroided MS^2^ spectrum of *m/z* 373.0 [M+HCO_2_H-H]^-^ showing the fragmentation pattern of 2’-*O*-acetylsalicin (**3**). Figure S6: UV-HPLC chromatogram (*λ* = 200 nm) of SPE fraction F6 prepared from the methanol extract of *S. pentandra*. Figure S7: Semi-preparative UV-HPLC (*λ* = 200 nm) chromatogram of fraction F6-12. Figure S8: Centroided MS^2^ spectrum of *m/z* 507.0 showing the fragmentation pattern of 2’,6’-*O*-diacetylsalicortin (**4**). Figure S9: Semi-preparative UV-HPLC chromatogram of fraction F6-13 at 200 nm. Figure S10: Centroided MS^2^ spectrum of *m/z* 603.2 showing the fragmentation pattern of lasiandrin (**5**). Figure S11: UV-HPLC chromatogram of SPE fraction F7 at 200 nm. Figure S12: Semi-preparative UV-HPLC chromatogram of fraction F7-8 at 200 nm. Figure S13: Centroided MS^2^ spectrum of *m/z* 527.0 showing the fragmentation pattern of tremulacin (**6**). Figure S14: Semi-preparative UV-HPLC chromatogram of fraction F5-2 at 252 nm. Figure S15: Centroided MS^2^ spectrum of *m*/*z* 441.1 showing the fragmentation pattern of cinnamrutinose A (**7**). Figure S16: Absolute configuration of the isolated salicylates, **1**-**2** and **4**-**6**, substituted with a HCH moiety determined by CD-spectroscopy. Figure S17: Semi-preparative UV-HPLC (*λ* = 200 nm) chromatogram of fraction F7-4. Table S1: ^13^C-NMR data of **1**-**6**. Table S2: ^1^H-NMR data of **1**-**6**. Table S3: Multiplicity and coupling constants of **1**-**6**. Table S4: NMR data (500/125 MHz) of **7**. Table S5: NMR data (500/125 MHz) of first diastereomer **8** (compound B) in methanol-*d_4_*. Table S6: NMR data (500/125 MHz) of second diastereomer **8** (compound C) in methanol-*d_4_*. Further materials regarding the isolation and identification (UV, MS, NMR, and CD data) of the bioactive substances can be found in the Supplementary Materials.
