# Supplementary material for: Identification of Salicylates in Willow Bark (Salix Cortex) for Targeting Peripheral Inflammation
Source: Int J Mol Sci. 2021 Oct 15;22(20):11138. doi: 10.3390/ijms222011138 (PMC8540557; doi:10.3390/ijms222011138)
Supplement: Supplementary file 1 [file ijms-22-11138-s001.zip › ijms-1410266-supplementary.pdf]

Supplementary Material

Identification of salicylates in willow bark (*Salix* cortex) for targeting peripheral inflammation

Kyriaki Antoniadou[1,†], Corinna Herz[2,†], Nguyen Phan Khoi Le[2], Verena Karolin Mittermeier-Kleßinger[1], Nadja Förster[3], Matthias Zander[3], Christian Ulrichs[3], Inga Mewis[3], Thomas Hofmann[1], Corinna Dawid[1,*], Evelyn Lamy[2,*]

[1] Chair of Food Chemistry and Molecular Sensory Science, Technical University of Munich, 85354 Freising, Germany; kyriaki.antoniadou@tum.de (K.A.); verena.mittermeier@tum.de (V.K.M.-K.); thomas.hofmann@tum.de (T.H.)
[2] Molecular Preventive Medicine, University Medical Center and Faculty of Medicine, University of Freiburg, 79108 Freiburg, Germany; corinna.herz@uniklinik-freiburg.de (C.H.); phan.khoi.nguyen.le@uniklinik-freiburg.de (N.P.K.L.);
[3] Urban Plant Ecophysiology, Humboldt University of Berlin, 14195 Berlin, Germany; nadja.foerster@hu-berlin.de (N.F.); matthias.zander@hu-berlin.de (M.Z.); christian.ulrichs@hu-berlin.de (C.U.); inga@entomology.de (I.M.)
[*] Correspondence: corinna.dawid@tum.de (C.D.); evelyn.lamy@uniklinik-freiburg.de (E.L.); Tel.: +49-8161/71-2949 (C.D.); +49-761/270-82150 (E.L.)
[†] These authors contributed equally

* To whom correspondence should be addressed

Evelyn Lamy, Prof. Dr.
Phone: +49 761 / 270-82150
Fax: +49 761 / 270-82030
email: evelyn.lamy@uniklinik-freiburg.de

Corinna Dawid, Prof. Dr.
Phone: +49 8161 / 71 2901
Fax: +49 8161 / 71 2949
email: corinna.dawid@tum.de

Table of contents

1. High-Performance Liquid Chromatography (HPLC) 3

2. Isolation and identification of compounds 1-3 and 7 from SPE fraction F5 3

3. Isolation and identification of compounds 4 and 5 from SPE fraction F6 6

4. Isolation and identification of compound 6 from SPE fraction F7 8

5. Isolation and identification of compound 7 from subfraction F5-2 9

6. QTRAP-LC-MS/MS conditions for sugar determination 10

7. Absolute configuration of salicylates 11

8. Purification of fraction F7-4 11

9. NMR spectroscopic data 13

1. High-Performance Liquid Chromatography (HPLC)

For gradient and method development by means of analytical HPLC, the analytical 250 x 4.6 mm Luna® phenyl-hexyl column was used at room temperature. Therefore, SPE fractions F5, F6, and F7 were diluted in ACN/H_2O (v/v 70/30) to 1 mg/mL and injected into the HPLC system using the autosampler. The detection was performed by ELSD (evaporative light-scattering detector) and DAD (diode-array detector). Since all compounds were visible by UV, no ELSD was further used. Following settings were applied: 200 nm wavelength, 30 µL injection volume, and 1 mL/min flow rate. For chromatographic separation 0.1% formic acid in water (solvent A) and 0.1% formic acid in acetonitrile (solvent B) were used starting with a mixture of 5% B for 5 min, then increasing eluent B to 100% within 55 min, held isocratic for another 5 min, decreased again to 5% B in 4 min, and finally held at 5% B for 6 min.

Preparative HPLC separation of the three SPE fractions was performed on a preparative 250 x 21.2 mm Luna® phenyl-hexyl column using UV-detection at 200 nm, flow rate of 20 mL/min, and the same solvents as mentioned above. SPE fractions F5 and F7 were diluted in pure methanol to a final concentration of 10 mg/mL, whereas the concentration of F6 was 5 mg/mL in methanol. Semi-preparative HPLC fractionation was performed on a semi-preparative 250 x 10 mm, 5 µm, Luna® pentafluorophenyl column (Phenomenex Ltd., Aschaffenburg, Deutschland) for fractions F5-5, F5-2 and F7-8, and on a semi-preparative 250 x 10 mm, 5 µm, Luna® phenyl-hexyl column (Phenomenex Ltd., Aschaffenburg, Germany) for fractions F6-12 and F6-13 using 0.1% formic acid in water (solvent A) and 0.1% formic acid in methanol (solvent B). Following conditions were applied for the isolation and purification (purity ≥ 95% by NMR) of the compounds **1**-**7** by means of preparative (flow rate of 20 mL/min) and semi-preparative (flow rate of 4.7 mL/min) HPLC:

2. Isolation and identification of compounds 1-3 and 7 from SPE fraction F5

For the purification of the compounds **1**-**3** and **7**, SPE fraction F5 was separated by means of preparative UV-HPLC (λ = 200 nm, injection volume: 500 µL, run time: 33 min). The chromatography started at 22% B, held for 3 min, increased in 10 min to 23.5% B, continued for 15 min at isocratic flow, and decreased to the initial conditions of 22% B, and finally held at 22% B for 3 min. Monitoring the UV signal at 200 nm, six fractions, F5-1 to F5-6, were collected, the solvent was removed, and used for further separation.

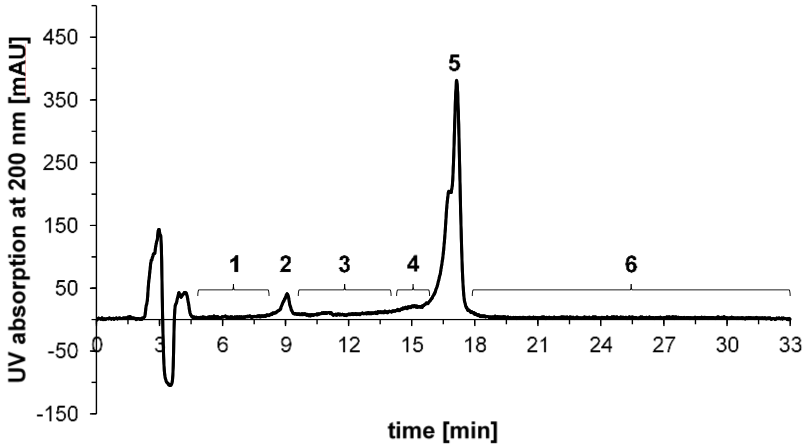

Figure S1: Preparative UV-HPLC chromatogram of SPE fraction F5 at 200 nm.

Further, fraction F5-5 (5 mg/mL in H_2O) was purified by means of semi-preparative UV-HPLC (λ = 200 nm, injection volume: 300 µL, run time: 65 min) and the following gradient: starting at 22% solvent B for 3 min, increased in 15 min to 27.7% B, held at 27.7% B for 2 min, increased to 52% B in 10 min, held at 52% B for 3 min, then increased further in 20 min to 57% B, held there for 2 min, and finally decreased in 3 min to 22% B, and held at 22% B for 2 min. 2′-*O*-acetylsalicortin (**1**), 3′-*O*-

acetylsalicortin (**2**) and 2′-*O*-acetylsalicin (**3**) were structurally elucidated in fractions F5-5-7 (**1**), F5-5-5 (**2**), and F5-5-3 (**3**) using LC-ToF-MS and 1D/2D-NMR experiments.

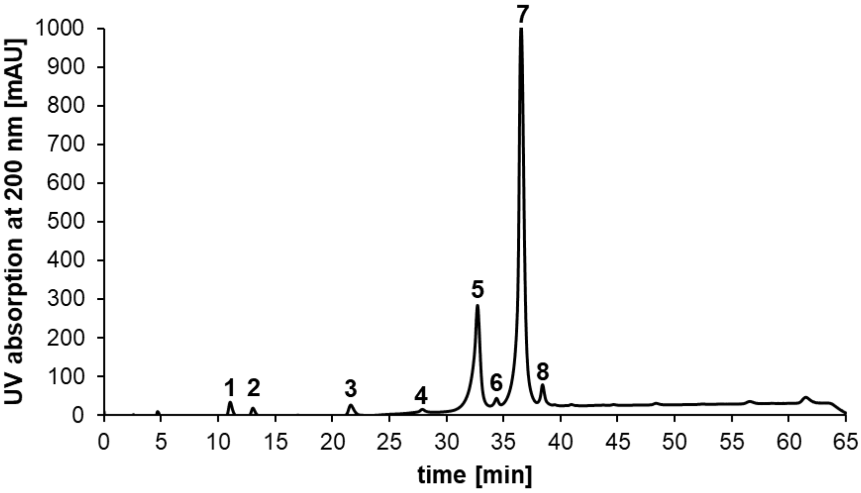

Figure S2: Semi-preparative UV-HPLC chromatogram of fraction F5-5 at 200 nm.

2′-*O*-acetylsalicortin (1)

$C_{22}H_{26}O_{11}$; UV (MeOH/H_2O, 1/1 v/v): λ_{max}=204, 220, 272 nm; LC-TOF-MS (ESI$^-$): *m/z* 511.1455 [M+HCO_2H-H]$^-$, 465.1434 [M-H]$^-$; LC-MS/MS (DP = -160 V, CE = -80 V): *m/z* (%) 154.88 (100), 136.86 (78), 122.86 (57), 120.90 (22), 92.95 (22), 82.99 (19), 80.99 (10).

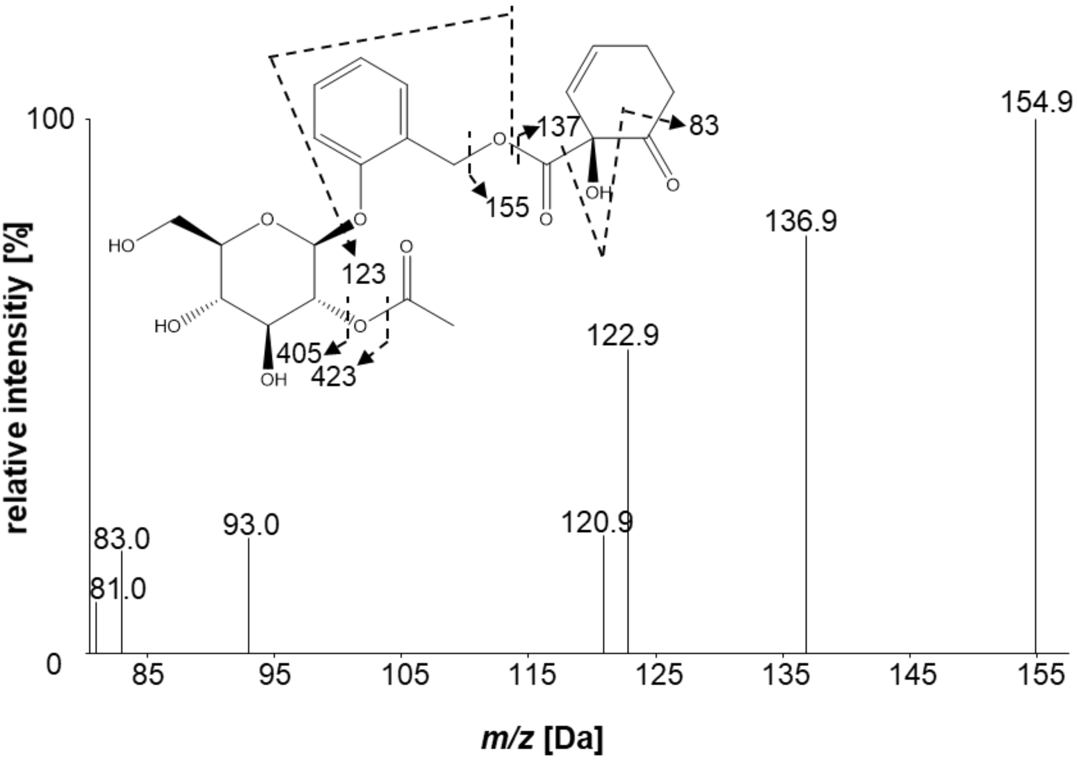

Figure S3: Centroided MS^2 spectrum of 464.9 Da showing the fragmentation pattern of 2′-*O*-acetylsalicortin (**1**).

3′-*O*-acetylsalicortin (2)

$C_{22}H_{26}O_{11}$; UV (MeOH/H_2O, 1/1 v/v): λ_{max}=200, 220, 272 nm; LC-TOF-MS (ESI$^-$): *m/z* 511.1458 [M+HCO_2H-H]$^-$, 465.1421 [M-H]$^-$; LC-MS/MS (DP = -80 V, CE = -66 V): *m/z* (%) 404.92 (41), 154.92 (100), 136.94 (76), 122.91 (61), 120.94 (19), 83.02 (19), 80.97 (5).

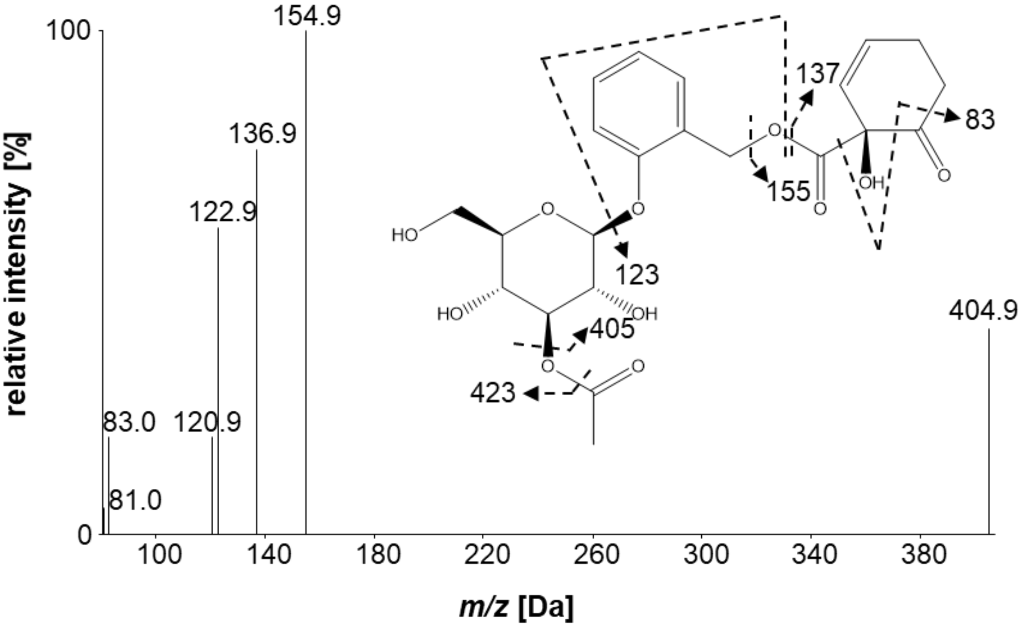

Figure S4: Centroided MS^2 spectrum of 465.0 Da showing the fragmentation pattern of 3′-*O*-acetylsalicortin (**2**).

2′-*O*-acetylsalicin (3)

$C_{15}H_{20}O_8$; UV (H_2O): λ_{max}= 204, 220, 268 nm; LC-TOF-MS (ESI): *m/z* 373.1130 [M+HCO_2H-H]$^-$, 327.1070 [M-H]$^-$; LC-MS/MS (DP = -5 V, CE = -76 V): *m/z* (%) 326.92 (100), 304.73 (73), 174.82 (32), 122.95 (87), 120.93 (8), 92.92 (3).

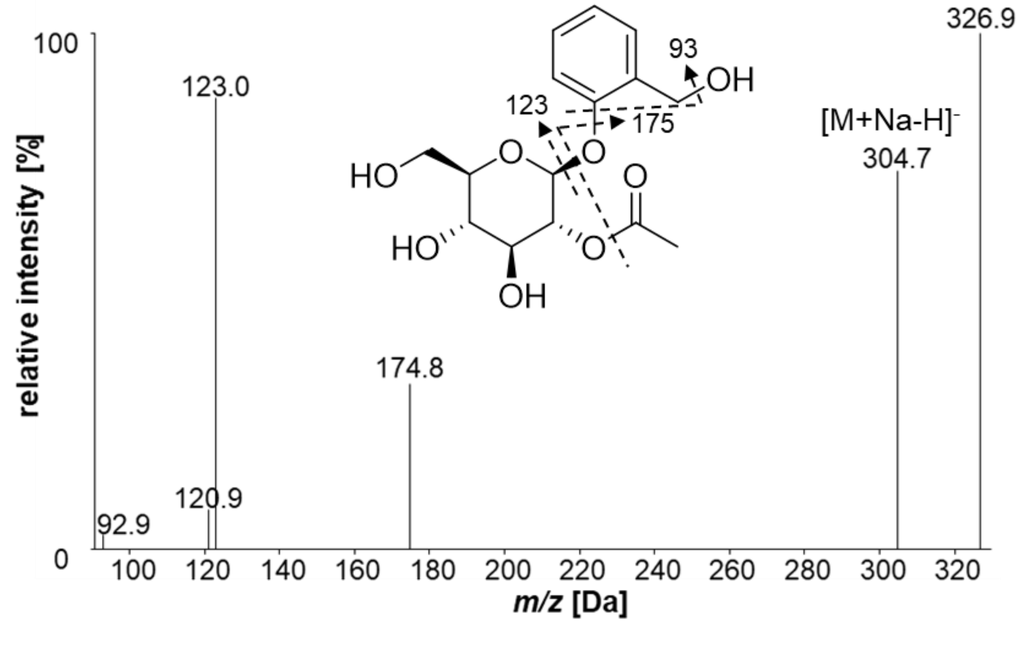

Figure S5: Centroided MS^2 spectrum of *m/z* 373.0 [M+HCO_2H-H]$^-$ showing the fragmentation pattern of 2′-*O*-acetylsalicin (**3**).

3. Isolation and identification of compounds 4 and 5 from SPE fraction F6

SPE Fraction F6 was purified by means of preparative UV-HPLC (λ = 200 nm, injection volume: 600 µL, run time: 35 min) and the following conditions: starting at 23% B, held isocratically for 3 min, increased in 21 min to 33% B, held at 33% B for 6 min, and decreased finally in 3 min to 23% B, which was held for 2 min. Fourteen fractions, F6-1 to F6-14, were collected, freed from the solvent, and used for further separation.

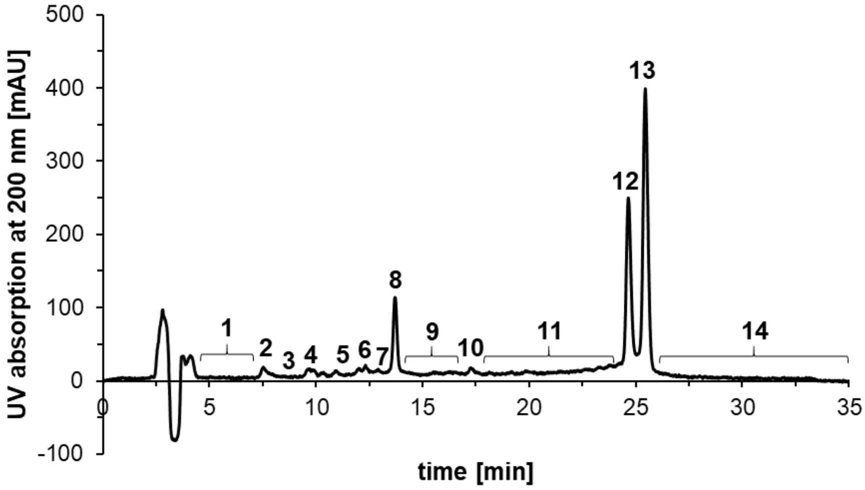

Figure S6: UV-HPLC chromatogram (λ = 200 nm) of SPE fraction F6 prepared from the methanol extract of *S. pentandra.*

Fraction F6-12 (10 mg/mL in MeOH/H2O (v/v 1/1)) was purified by means of semi-preparative UV-HPLC (λ = 200 nm, injection volume: 150 µL, run time: 50 min): the chromatography started at 20% B, held there for 3 min, increased in 43.5 min to 32% B, decreased again in 1.5 min to 20% B and held at 20% B for 2 min. 2′,6′-*O*-diacetylsalicortin (**4**) was structurally elucidated in fraction 6-12-2 using LC-ToF-MS and 1D/2D-NMR experiments.

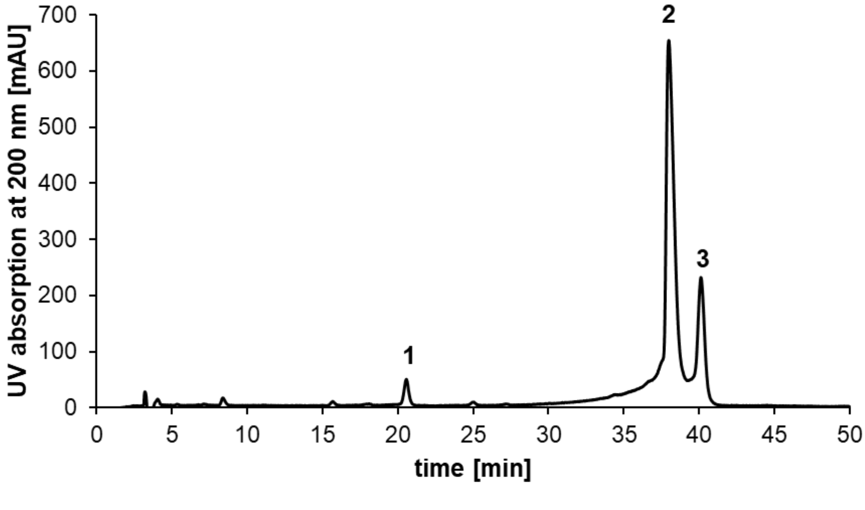

Figure S7: Semi-preparative UV-HPLC (λ = 200 nm) chromatogram of fraction F6-12.

2′,6′-*O*-diacetylsalicortin (4)

$C_{24}H_{28}O_{12}$; UV (MeOH/H_2O, 1/1 v/v): λ_{max}=200, 272, 300 nm; LC-TOF-MS (ESI$^-$): *m/z* 553.1569 [M+HCO_2H-H]$^-$, 507.1551 [M-H]$^-$; LC-MS/MS (DP = -160 V, CE = -62 V): *m/z* (%) 155.00 (81), 136.90 (100), 122.89 (58), 120.91 (22), 92.94 (23), 82.94 (17), 80.98 (4).

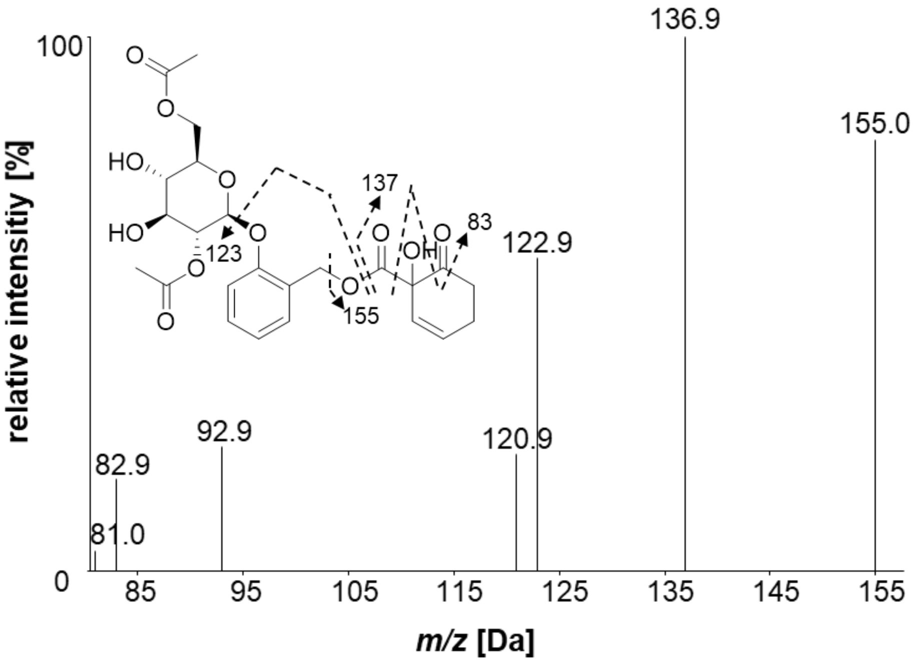

Figure S8: Centroided MS² spectrum of *m/z* 507.0 showing the fragmentation pattern of 2′,6′-*O*-diacetylsalicortin (**4**).

Fraction F6-13 (10 mg/mL in MeOH/H2O (v/v 1/1)) was purified by means of semi-preparative UV-HPLC (λ = 200 nm, injection volume: 200 µL, run time: 34 min) and the following gradient: starting with an isocratic step at 27% B for 3 min, increased in 27 min to 32% B, finally decreased in 4 min to 27% B and held there. Lasiandrin (**5**) was structurally elucidated in fraction 6-13-2 using LC-ToF-MS and 1D/2D-NMR experiments.

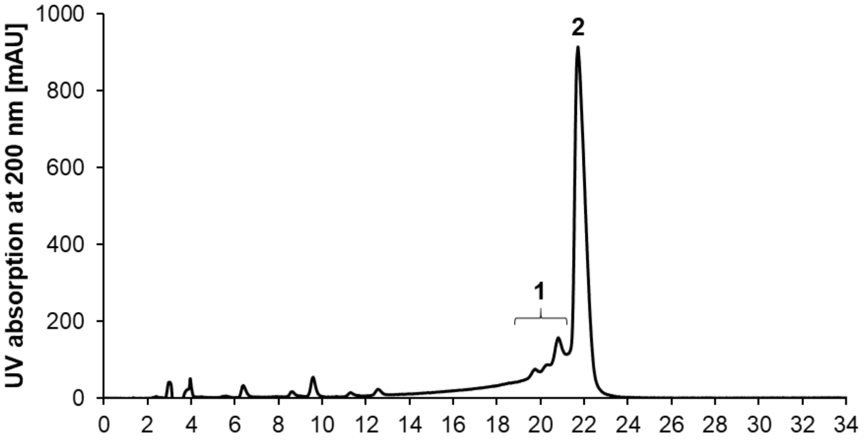

Figure S9: Semi-preparative UV-HPLC chromatogram of fraction F6-13 at 200 nm.

lasiandrin (5)

$C_{29}H_{32}O_{14}$; UV (MeOH/H_2O, 1/1 v/v): λ_{max}=200, 272, 300 nm; LC-TOF-MS (ESI$^-$): *m/z* 649.1791 [M+HCO_2H-H]$^-$, 603.1762 [M-H]$^-$; LC-MS/MS (DP = -135 V, CE = -84 V): *m/z* (%) 464.99 (100), 154.90 (85), 136.91 (83), 110.95 (32), 108.93 (15), 92.95 (30), 82.98 (26), 80.96 (10).

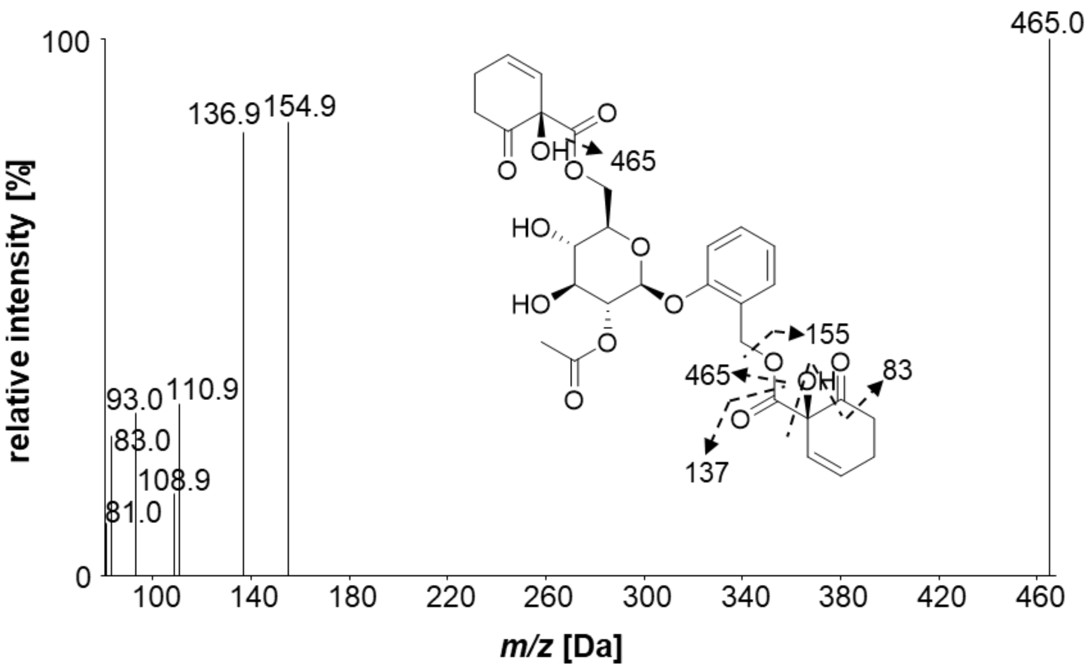

Figure S10: Centroided MS^2 spectrum of *m/z* 603.2 showing the fragmentation pattern of lasiandrin (**5**).

4. Isolation and identification of compound 6 from SPE fraction F7

SPE fraction F7 was purified by means of preparative UV-HPLC (λ = 200 nm, injection volume: 700 µL, run time: 34 min): the chromatography started at 28% B, held there for 3 min, increased in 7 min to 32% B, isocratic for 2 min, increased in 14 min to 37% B, isocratic at 37% B for 2 min, decreased again in 3 min to 28% B, where it was held isocratic for 3 min. Fourteen fractions, F7-1 to F7-14, were collected, freed from the solvent, and used for further separation.

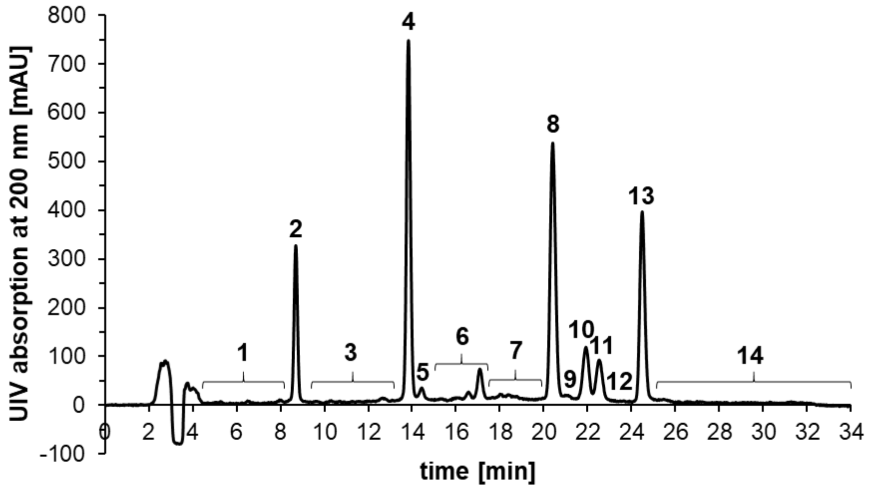

Figure S11: UV-HPLC chromatogram of SPE fraction F7 at 200 nm.

Fraction F7-8 was purified by means of semi-preparative UV-HPLC (λ = 200 nm, injection volume: 200 µL, run time: 42 min) and the following chromatographic conditions: starting at 26% B, held there isocratic for 3 min, increased in 23 min to 62% B, held at 62% B for 10 min, decreased in 4 min to 26 % B, where it was held isocratic for 2 min. Tremulacin (**6**) was structurally characterized in fraction 7-8-4 using LC-ToF-MS and 1D/2D-NMR experiments.

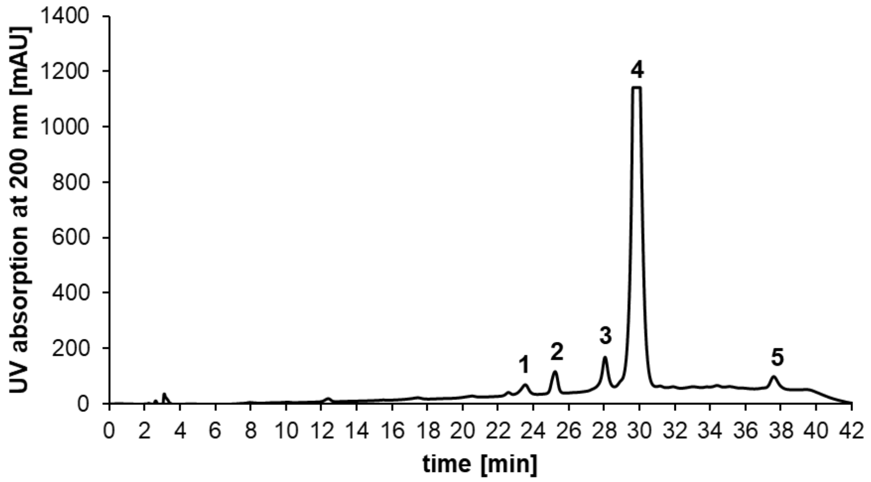

Figure S12: Semi-preparative UV-HPLC chromatogram of fraction F7-8 at 200 nm.

tremulacin (6)

$C_{27}H_{28}O_{11}$; UV (MeOH/H_2O, 1/1 v/v): λ_{max}=204, 236, 272 nm; LC-TOF-MS (ESI$^-$): *m/z* 573.1617 [M+HCO_2H-H]$^-$, 527.1586 [M-H]$^-$; LC-MS/MS (DP = -20 V, CE = -86 V): *m/z* (%) 404.95 (100), 154.90 (49), 136.90 (39), 122.92 (14), 120.94 (67), 82.97 (10), 80.97 (10), 76.97 (22).

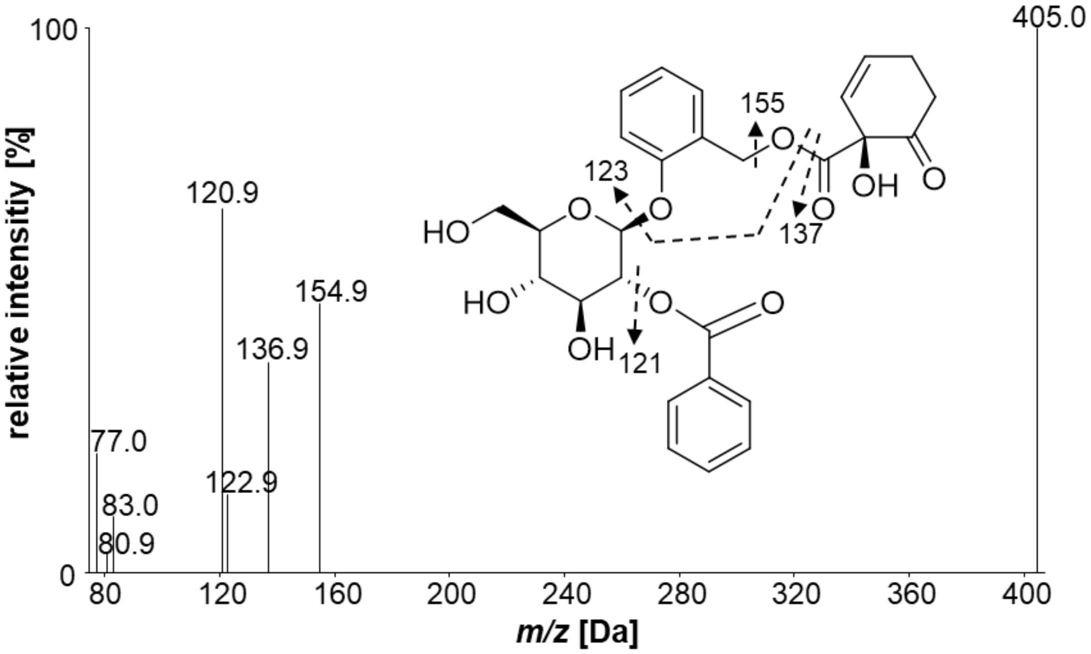

Figure S13: Centroided MS^2 spectrum of *m/z* 527.0 showing the fragmentation pattern of tremulacin (**6**).

5. Isolation and identification of compound 7 from subfraction F5-2

Fraction F5-2 (1 mg/mL in methanol/water (v/v 1/1)) was purified by means of semi-preparative UV-HPLC (λ = 252 nm, injection volume: 300 µL, run time: 37 min) using the following chromatographic conditions: starting at 22% B, held there for 3 min, increased in 17 min to 45% B, held at 45% B for 2 min, increased further in 8 min to 50% B, held at 50% B for 3 min, decreased in 2 min to 22% B, and finally held isocratic for 2 min. Cinnamrutinose A was structurally elucidated in fraction 5-2-2 (**7**) using LC-ToF-MS and 1D/2D-NMR experiments.

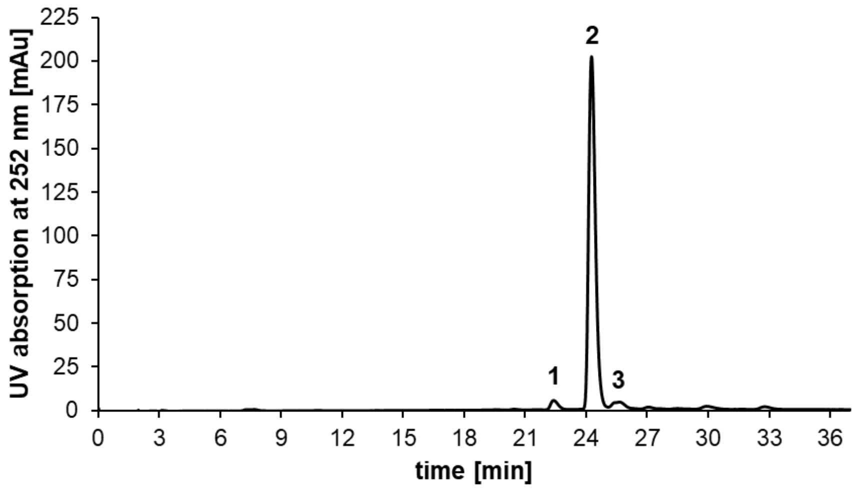

Figure S14: Semi-preparative UV-HPLC chromatogram of fraction F5-2 at 252 nm.

cinnamrutinose A (7)
$C_{21}H_{30}O_{10}$; UV (MeOH/H_2O, 1/1 v/v): λ_{max}=204, 252 nm; LC-TOF-MS (ESI$^-$): *m/z* 487.1824 [M+HCO_2H-H]$^-$, 441.1768 [M-H]$^-$; LC-MS/MS (DP = -55 V, CE = -56 V): *m/z* (%) 306.92 (100), 162.92 (41), 160.89 (3), 126.91 (1), 124.91 (14), 118.90 (13), 102.92 (19), 100.87 (7), 58.97 (4).

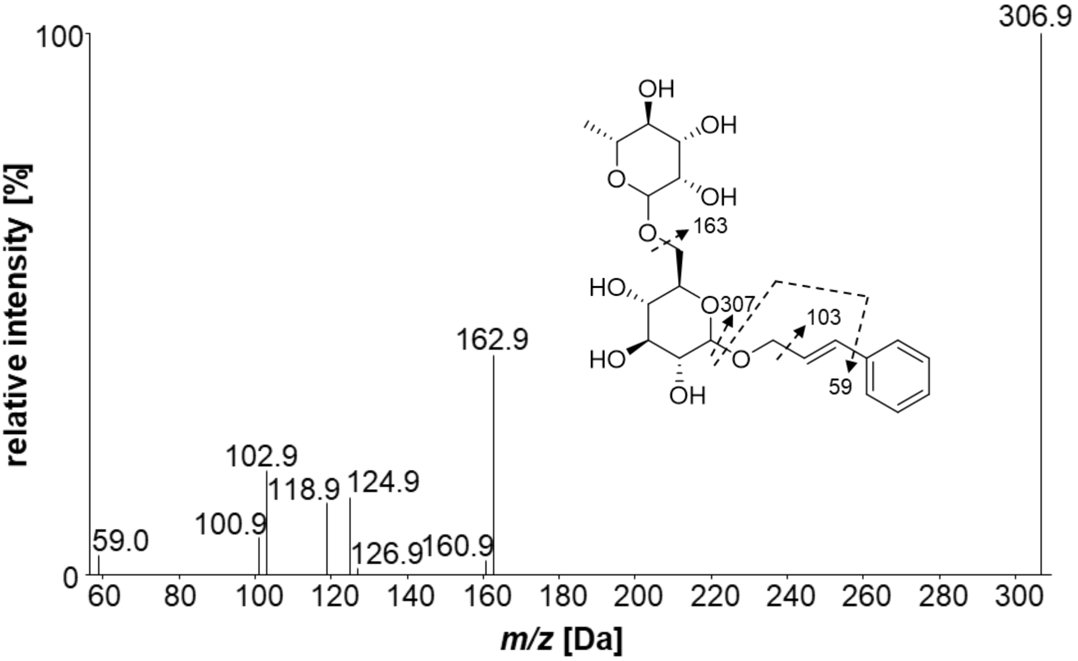

Figure S15: Centroided MS^2 spectrum of *m/z* 441.1 showing the fragmentation pattern of cinnamrutinose A (**7**).

6. QTRAP-LC-MS/MS conditions for sugar determination

For the determination of the sugar residues, the QTRAP-LC-MS/MS system was employed using 1% formic acid in H_2O (solvent A) and 1% formic acid in acetonitrile (solvent B) and positive ionization mode (ESI$^+$). For chromatography a 2.1x100 mm, 1.7 µm, Kinetex F5 column (Phenomenex, Aschaffenburg, Germany) was used and the following gradient (adapted from Schmid et al. (2018)) was applied: starting conditions were 5% of solvent B, held isocratic for 1.99 min at 5% B, then, increased to 20% in 3 min, in 21 min to 25% and in 1 min to 100 % B, held isocratic for 3 min at 100% B and decreased to 5% B in 1 min and finally held isocratic for 4 min at 5% B.

The MRM transitions Q1/Q3 of the derivatized sugars *D*-glucose (*m/z* 461.0/298.1), *L*-glucose (*m/z* 461.0/298.1), *D*-galactose (*m/z* 461.1/298.2), *D*-galacturonic acid (*m/z* 475.0/312.1), *D*-glucuronic acid (*m/z* 475.0/312.1), and *L*-rhamnose (*m/z* 445.0/282.1), as well as the DP, CE, and CXP values were adopted from Schmid et al. (2018).

7. Absolute configuration of salicylates

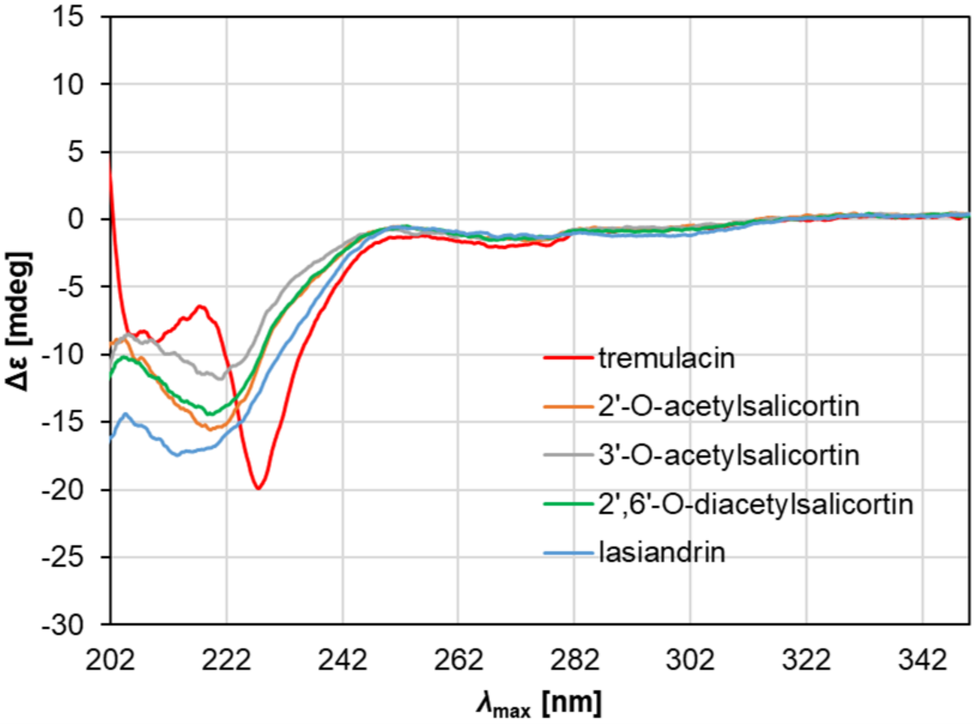

Figure S16: Absolute configuration of the isolated salicylates, 1-2 and 4-6, substituted with a HCH moiety determined by CD-spectroscopy. 2′-*O*-acetylsalicortin (1): Δε = -15.5 mdeg (*c* 0.43 mM, CH_3OH, λ_{max} = 220 nm), 3′-*O*-acetylsalicortin (2): Δε = -11.7 mdeg (*c* 0.43 mM, CH_3OH, λ_{max} = 221 nm), 2′,6′-*O*-diacetylsalicortin (4): Δε = -14.4 mdeg (*c* 0.39 mM, CH_3OH, λ_{max} = 220 nm), lasiandrin (5): Δε = -17.2 mdeg (*c* 0.33 mM, CH_3OH, λ_{max} = 216 nm), tremulacin (6): Δε = -19.6 and -8.8 mdeg (*c* 0.38 mM, CH_3OH, λ_{max} = 228, 209 nm).

8. Purification of fraction F7-4

For the semi-preparative UV-HPLC (λ = 200 nm) fractionation of F7-4 (15 mg/mL in methanol) a semi-preparative 250 x 10 mm, 5 µm, PFP column was used. The chromatographic separation was performed using 0.1 % formic acid in water (solvent A) and 0.1 % formic acid in methanol (solvent B). The injection volume for both fractions was 100 µL. The run time of the chromatographic separation of fraction F7-4 was 56 min. Chromatography of F7-4 started at 40% B, held at 40% B for 3 min, increased to 53% B in 17 min, held for 2 min, increased further to 57% B in 26 min, held for 2 min, and finally decreased again to 40% B in 3 min, which was held isocratic for 3 min. *β*-D-Glucopyranoside, 2-[[[(1-hydroxy-6,6dihydroxy-2-cyclohexen-1-yl)dihydroxy]oxy]methyl]phenyl, 2-acetate (**8**)was structurally elucidated in fraction F7-4-6 using LC-ToF-MS and 1D/2D-NMR experiments.

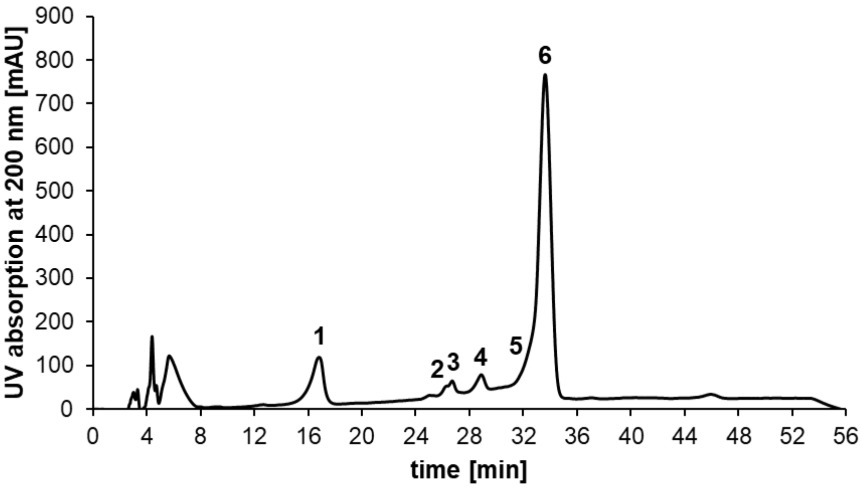

Figure S17: Semi-preparative UV-HPLC (λ = 200 nm) chromatogram of fraction F7-4.

***β*-D-Glucopyranoside, 2-[[[(1-hydroxy-6,6-dihydroxy-2-cyclohexen-1-yl)dihydroxy]oxy]methyl]phenyl, 2-acetate (8) in F7-4-6**
$C_{22}H_{28}O_{12}$; LC-TOF-MS (ESI^-): *m/z* 465.1401 $[M-H_2O-H]^-$

After acetalization:
$C_{28}H_{36}O_{12}$; LC-TOF-MS (ESI^-): *m/z* 563.2126 $[M+\text{two acetal groups}-H]^-$

9. NMR spectroscopic data

The spectroscopic data of the isolated compounds are shown in Table 1-6. For NMR analysis, compound **6** was dissolved in DMSO-*d*6, **1**, **2**, **4**, and **5** in acetone-*d*6, **6** in acetonitrile-*d*3, and **3** in methanol-*d*4.

Table S1: ^{13}C-NMR data of **1**-**6**. *compound 5: C(8) = [C=O], **compound 6: C(1′′) = [C=O], C(2′′) = [C].

Position	HSQC	δC [ppm]					
		1	2	3	4	5	6
1	[C]	155.88	156.41	155.85	155.78	155.85	154.20
2	[C]	125.94	125.83	131.92	126.10	126.17	124.34
3	[CH]	129.50	130.10	128.90	129.61	130.61	127.94
4	[CH]	123.36	123.10	123.70	123.56	123.66	122.30
5	[CH]	130.40	130.66	129.47	130.35	129.55	129.40
6	[CH]	116.49	116.35	115.96	116.62	116.80	115.05
7	[CH2]	63.08	63.67	59.97	63.07	62.17	61.40
8	[C=O]	170.79	170.86	-	170.78	170.78	169.86
9	[C]	78.81	78.83	-	78.85	78.86	77.35
10	[CH]	129.30	129.29	-	129.31	129.30	128.66
11	[CH]	132.43	132.44	-	132.41	132.43	131.56
12	[CH2]	27.20	27.17	-	27.20	27.20	25.85
13	[CH2]	36.09	36.11	-	36.06	36.07	35.56
14	[C=O]	206.20	206.21	-	206.20	206.18	205.92
1′	[CH]	100.18	102.03	100.75	100.06	100.25	98.41
2′	[CH]	74.39	72.87	75.07	74.26	74.41	73.79
3′	[CH]	75.66	77.70	75.98	75.06	75.45	74.29
4′	[CH]	71.44	69.36	71.41	71.47	71.39	69.89
5′	[CH]	78.05	78.46	78.35	75.50	75.05	77.28
6′	[CH2]	62.37	62.19	62.40	64.01	65.76	60.54
7′	[C=O]	-	-	-	170.92	-	-
8'	[CH3]	-	-	-	20.72	170.78*	-
9′	[C]	-	-	-	-	78.82	-
10′	[CH]	-	-	-	-	129.26	-
11′	[CH]	-	-	-	-	132.35	-
12′	[CH2]	-	-	-	-	27.20	-
13′	[CH2]	-	-	-	-	36.07	-
14′	[C=O]	-	-	-	-	206.18	-
1′′	[CH3]	21.02	21.11	21.02	21.00	21.01	165.10**
2′′	[C=O]	170.30	170.79	170.55	170.27	170.26	129.74**
3′′	[CH]	-	-	-	-	-	129.34
4′′	[CH]	-	-	-	-	-	128.68
5′′	[CH]	-	-	-	-	-	133.38
6′′	[CH]	-	-	-	-	-	128.68
7′′	[CH]	-	-	-	-	-	129.34

Table S2: ^{1}H-NMR data of **1-6**. *compound 5: C(8) = [C=O], ** compound 6: C(1″) = [C=O], C(2″) = [C].

Position	HSQC	δ_H [ppm]					
		**1**	**2**	**3**	**4**	**5**	**6**
1	[C]	-	-	-	-	-	-
2	[C]	-	-	-	-	-	-
3	[CH]	7.31	7.33	7.38	7.33	7.37	7.13
4	[CH]	7.05	7.05	7.03	7.06	7.08	7.00
5	[CH]	7.28	7.31	7.21	7.31	7.32	7.29
6	[CH]	7.21	7.25	7.13	7.22	7.22	7.18
7	[CH_2]	5.18	5.23<br>5.35	4.55	5.13	5.18<br>5.12	4.80<br>4.95
8	[C=O]	-	-	-	-	-	-
9	[C]	-	-	-	-	-	-
10	[CH]	5.80	5.78	-	5.80	5.80	5.66
11	[CH]	6.14	6.13	-	6.14	6.14	6.07
12	[CH_2]	2.49-2.54	2.48-2.53	-	2.48-2.53	2.46-2.54	2.39-2.46
		2.67-2.76	2.64-2.71		2.66-2.75	2.62-2.73	2.47-2.56
13	[CH_2]	2.59-2.66	2.54-2.57	-	2.54-2.58	2.53-2.59	2.47-2.52
		2.94-2.86	2.83-2.90		2.85-2.93	2.84-2.92	2.69-2.63
14	[C=O]	-	-	-	-	-	-
1′	[CH]	5.13	5.11	5.05	5.16	5.17	5.29
2′	[CH]	5.02	3.64	5.03	5.06	5.02	5.05
3′	[CH]	3.72	5.08	3.66	3.82	3.74	3.69
4′	[CH]	3.57	3.66	3.50	3.58	3.52	3.34
5′	[CH]	3.58	3.63	3.51	3.77	3.85	3.51
6′	[CH_2]	3.73	3.74	3.71	3.74	4.27	3.55
		3.91	3.89	3.91	3.91	4.64	3.76
7′	[C=O]	-	-	-	-	-	-
8'	[CH_3]	-	-	-	2.03	-*	-
9′	[C]	-	-	-	-	-	-
10′	[CH]	-	-	-	-	5.75	-
11′	[CH]	-	-	-	-	6.08	-
12′	[CH_2]	-	-	-	-	2.46-2.54<br>2.62-2.73	-
13′	[CH_2]	-	-	-	-	2.53-2.59<br>2.84-2.92	-
14′	[C=O]	-	-	-	-	-	-
1′′	[CH_3]	2.07	2.05	2.12	2.10	2.08	-**
2′′	[C=O]	-	-	-	-	-	-**
3′′	[CH]	-	-	-	-	-	7.98
4′′	[CH]	-	-	-	-	-	7.52
5′′	[CH]	-	-	-	-	-	7.65
6′′	[CH]	-	-	-	-	-	7.52
7′′	[CH]	-	-	-	-	-	7.98

Table S3: Multiplicity and coupling constants of **1-6**. *compound 5: C(8) = [C=O], ** compound 6: C(1′′) = [C=O], C(2′′) = [C].

Position	HSQC	Multiplicity; *J* [Hz]					
		**1**	**2**	**3**	**4**	**5**	**6**
1	[C]	-	-	-	-	-	-
2	[C]	-	-	-	-	-	-
3	[CH]	m	m	m	m	m	dd; 7.70, 1.51
4	[CH]	td; 7.50, 1.08	td; 7.50, 1.15	m	td; 7.44, 1.12	td; 7.42, 1.00	td; 7.62,1.00
5	[CH]	m	m	m	m	dd; 7.78, 1.73	ddd; 7.97, 1.52
6	[CH]	d; 8.00	d; 7.84	dd; 8.20, 1.05	dd; 8.78, 1.06	d; 8.19	d; 8.21
7	[CH_2]	d; 2.73 (overlap)	d; 12.80<br>d; 12.80	q; 15.46, 13.60 (overlap)	d; 13.07 (overlap)	d; 12.87 (overlap)<br>d; 12.87	d; 13.37<br>d; 13.37
8	[C=O]	-	-	-	-	-	-
9	[C]	-	-	-	-	-	-
10	[CH]	dt; 9.70, 1.70	dt; 9.70, 1.70	-	dt; 9.81, 3.48	dt; 9.80, 1.70	dt: 9.90, 1.57
11	[CH]	dt; 9.70, 3.80	dt; 9.70, 3.80	-	dt; 9.67, 3.71	dt; 9.81, 3.96	dt; 9.81, 3.74
12	[CH_2]	m<br>m	m<br>m	-	m<br>m	m<br>m	m<br>m (overlap)
13	[CH_2]	m<br>m	m<br>m	-	m<br>m	m<br>m	m (overlap)<br>m
14	[C=O]	-	-	-	-	-	-
1′	[CH]	d; 7.16	d; 7.70	d; 8.05	d; 7.16	d; 8.19	d; 8.13
2′	[CH]	dd; 8.08, 1.62	m	dd: 8.01, 1.46	dd; 8.33, 1.58	dd; 8.10, 1.55	dd; 8.08, 1.62
3′	[CH]	m	d; 9.30	m	m	t; 9.45	td; 5.49, 3.74
4′	[CH]	m	m	m	t; 9.31	t; 9.45	s
5′	[CH]	m	m	m	t; 9.44	ddd; 6.90, 5.12, 2.04	m
6′	[CH_2]	m<br>m	dd; 11.35, 3.69<br>dd; 11.39, 2.99	m<br>m	dd; 11.75, 2.30<br>dd; 11.95, 6.24	dd; 11.80, 6.79<br>dd; 11.82, 2.06	m<br>dd; 10.68, 4.99
7′	[C=O]	-	-	-	-	-	-
*8′	[CH_3]	-	-	-	s	-	-
9′	[C]	-	-	-	-	-	-
10′	[CH]	-	-	-	-	dt; 9.80, 1.79	-
11′	[CH]	-	-	-	-	dt; 9.93, 3.89	-
12′	[CH_2]	-	-	-	-	m<br>m	-
13′	[CH_2]	-	-	-	-	m<br>m	-
14′	[C=O]	-	-	-	-	-	-
**1′′	[CH_3]	s	s	s	s	s	-
**2′′	[C=O]	-	-	-	-	-	-
3′′	[CH]	-	-	-	-	-	t; 7.94
4′′	[CH]	-	-	-	-	-	m
5′′	[CH]	-	-	-	-	-	m
6′′	[CH]	-	-	-	-	-	t; 7.94
7′′	[CH]	-	-	-	-	-	m

Table S4: NMR data (500/125 MHz) of **7**.

Position	HSQC	δC [ppm]	δH [ppm]	Multiplicity; *J* [Hz]
1	[CH_2]	70.11	4.24<br>4.43	ddd; 12.83, 6.54, 1.44<br>ddd; 12.80, 7.04, 1.52
2	[CH]	126.84	6.35	ddd; 16.14, 5.61
3	[CH]	133.01	6.69	dt; 15.89, 1.82
4	[C]	137.80	-	-
5	[CH]	127.41	7.44	d; 7.60
6	[CH]	129.67	7.35	m
7	[CH]	128.73	7.30	m
8	[CH]	129.67	7.35	m
9	[CH]	127.41	7.44	d; 7.60
1′	[CH]	102.85	4.31	d; 7.75
2′	[CH]	74.70	3.14	t; 8.10, 8.59
3′	[CH]	71.25	3.22	m
4′	[CH]	77.63	3.37	m
5′	[CH]	73.75	3.25	m
6′	[CH_2]	67.90	3.64<br>3.88	m<br>dd; 11.46, 1.92
1″	[CH]	101.66	4.72	dd; 16.35, 1.03
2″	[CH]	71.74	3.78	dd; 3.78, 1.53
3″	[CH]	72.26	3.54	dd; 9.45, 3.63
4″	[CH]	76.34	3.29	m
5″	[CH]	69.07	3.59	m
6″	[CH_3]	18.10	1.21	d; 6.41

Table S5: NMR data (500/125 MHz) of first diastereomer **8** (compound B) in methanol-d_4.

Position	HSQC	δ_C [ppm]	δ_H [ppm]	Multiplicity; *J* [Hz]
1	[C]	156.06	-	-
2	[C]	126.72	-	-
3	[CH]	129.67	7.39	m
4	[CH]	123.82	7.04	dt; 7.07, 1.14
5	[CH]	130.39	7.30	m
6	[CH]	116.85	7.21	ddd; 1.23, 7.57
7	[CH_2]	63.13	5.11-5.18	m
8	[C=O]	171.58	-	-
9	[C]	86.75	-	-
10	[CH]	127.19	5.59	dd; 10.10, 2.75
11	[CH]	131.12	6.02	m
12	[CH_2]	23.31	1.98-2.06	m
			2.17-2.24	m
13	[CH_2]	33.19	1.93-2.00	m
			2.41-2.53	m
14	[C]	104.47	-	-
1′	[CH]	100.74	5.09	m
2′	[CH]	75.00	5.03	m
3′	[CH]	75.98	3.64-3.67	m
4′	[CH]	71.42	3.49	m
5′	[CH]	78.38	3.50	m
6′	[CH_2]	62.41	3.75	m
			3.94	m
1′′	[CH_3]	21.10	2.15	s
2′′	[C=O]	171.98	-	-

Table S6: NMR data (500/125 MHz) of second diastereomer **8** (compound C) in methanol-d_4.

**Position**	**HSQC**	**δ_C [ppm]**	**δ_H [ppm]**	**Multiplicity; *J* [Hz]**
1	[C]	156.06	-	-
2	[C]	126.72	-	-
3	[CH]	129.67	7.39	m
4	[CH]	123.82	7.04	dt; 7.07, 1.14
5	[CH]	130.39	7.30	m
6	[CH]	116.85	7.21	ddd; 1.23, 7.57
7	[CH_2]	63.13	5.11-5.18	m
8	[C=O]	173.02	-	-
9	[C]	77.33	-	-
10	[CH]	128.44	5.53	dt (10.03, 2.01)
11	[CH]	132.80	5.98	dt (9.98, 3.62)
12	[CH_2]	25.29	2.19-2.26	m
			2.33-3.39	m
13	[CH_2]	32.13	2.44-2.51	m
14	[C]	111.81	-	-
1′	[CH]	100.74	5.09	m
2′	[CH]	75.00	5.03	m
3′	[CH]	75.98	3.64-3.67	m
4′	[CH]	71.42	3.49	m
5′	[CH]	78.38	3.50	m
6′	[CH_2]	62.41	3.75	m
			3.94	m
1′′	[CH_3]	21.10	2.15	s
2′′	[C=O]	171.98	-	-
